# Using the Synergy between HPLC-MS and MALDI-MS Imaging to Explore the Lipidomics of Clear Cell Renal Cell Carcinoma

**DOI:** 10.1021/acs.analchem.2c03953

**Published:** 2023-01-13

**Authors:** Lucía Martín-Saiz, Beatriz Abad-García, Jon D. Solano-Iturri, Lorena Mosteiro, Javier Martín-Allende, Yuri Rueda, Amparo Pérez-Fernández, Miguel Unda, Pedro Coterón-Ochoa, Aintzane Goya, Alberto Saiz, Jennifer Martínez, Begoña Ochoa, Olatz Fresnedo, Gorka Larrinaga, José A. Fernández

**Affiliations:** † Department of Physical Chemistry, Faculty of Science and Technology, University of the Basque Country (UPV/EHU), B. Sarriena, s/n, Leioa 48940, Spain; ‡ Central Analysis Service, Faculty of Science and Technology, University of the Basque Country (UPV/EHU), Leioa 48940, Spain; § Service of Anatomic Pathology, Donostia University Hospital, Donostia/San Sebastian 20014, Spain; ∥ Biocruces Bizkaia Health Research Institute, Barakaldo 48903, Spain; ⊥ Service of Anatomic Pathology, Cruces University Hospital, Barakaldo 48903, Spain; # Lipids & Liver, Department of Physiology, Faculty of Medicine and Nursing, University of the Basque Country (UPV/EHU), B. Sarriena, s/n, Leioa 48940, Spain; ∇ Service of Urology, Basurto University Hospital, Bilbao 48003, Spain; ○ Service of Urology, Galdakao-Usansolo University Hospital, Galdakao 48960, Spain; ◆ Service of Anatomic Pathology, Galdakao-Usansolo University Hospital, Galdakao 48960, Spain; ¶ Department of Nursing and Department of Physiology, Faculty of Medicine and Nursing (UPV/EHU), Leioa 48940, Spain

## Abstract

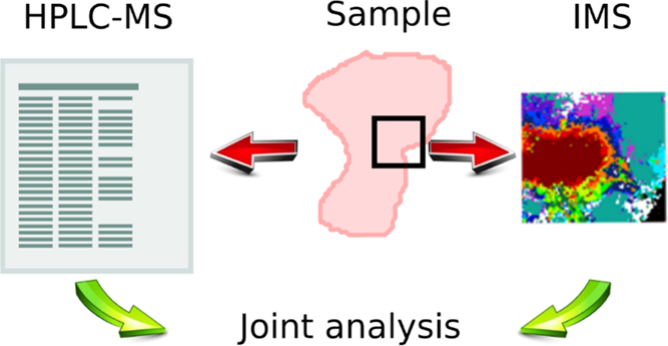

Lipid imaging mass spectrometry (LIMS) has been tested in several pathological contexts, demonstrating its ability to segregate and isolate lipid signatures in complex tissues, thanks to the technique’s spatial resolution. However, it cannot yet compete with the superior identification power of high-performance liquid chromatography coupled to mass spectrometry (HPLC-MS), and therefore, very often, the latter is used to refine the assignment of the species detected by LIMS. Also, it is not clear if the differences in sensitivity and spatial resolution between the two techniques lead to a similar panel of biomarkers for a given disease. Here, we explore the capabilities of LIMS and HPLC-MS to produce a panel of lipid biomarkers to screen nephrectomy samples from 40 clear cell renal cell carcinoma patients. The same set of samples was explored by both techniques, and despite the important differences between them in terms of the number of detected and identified species (148 by LIMS and 344 by HPLC-MS in negative-ion mode) and the presence/absence of image capabilities, similar conclusions were reached: using the lipid fingerprint, it is possible to set up classifiers that correctly identify the samples as either healthy or tumor samples. The spatial resolution of LIMS enables extraction of additional information, such as the existence of necrotic areas or the existence of different tumor cell populations, but such information does not seem determinant for the correct classification of the samples, or it may be somehow compensated by the higher analytical power of HPLC-MS. Similar conclusions were reached with two very different techniques, validating their use for the discovery of lipid biomarkers.

## Introduction

Since the introduction of imaging mass spectrometry (IMS),^1,2^ this technique has demonstrated a great potential to map the distribution of proteins, metabolites, and drugs in tissues of diverse origins.^3,4^ However, many are still pending tasks to bring IMS into clinics and to develop really useful tests for the early detection of pathologies, the diagnosis of difficult cases, or others.^5,6^

One of the main problems that IMS faces, at least in its matrix-assisted laser desorption/ionization (MALDI)-IMS variant, is the limited ability to identify detected species.^7^ As they are extracted directly from the tissue, it is not possible to introduce a separation stage as in high-performance liquid chromatography-mass spectrometry (HPLC-MS). For example, when applied to the field of lipidomics, ion suppression effect and the existence of many isobaric species complicate lipid identification, seriously hampering data interpretation.^8^ To circumvent this problem, some mass spectrometers incorporate an ion mobility cell that offers a limited discrimination power, at the expense of a serious reduction in acquisition speed. Nevertheless, important advances in that direction have been made in recent years.^9,10^

Another approach that most researchers use is combining HPLC-MS with lipid imaging mass spectrometry (LIMS) to create a library of lipids that improves identification: species that are not present in the library are usually discarded as possible candidates for the m/z in the spectra recorded directly from the tissue.^11^ When information from HPLC-MS is combined with on-tissue MS/MS or MS3, robust assignments for a substantial number of m/z may be obtained.^12^ Still, the problem of the different detectability of lipid classes persists, making it difficult to extract conclusions about their relative abundance.^13,14^

Here, we combine HPLC-MS with LIMS to evaluate the performance of both techniques for the classification of samples of a healthy kidney and clear cell renal cell carcinoma (ccRCC). This is the most frequent kidney neoplasia in humans. That is why several mass spectrometry studies have been published aiming at the classification of samples into tumoral and nontumoral, or into ccRCC and chromophobe renal cell carcinoma (ChRCC). For that purpose, the use of the peptidic and/or lipidic signature has been explored, both in fresh tissue and in paraffin-embedded tissue.^15−25^ However, most of those studies report a very limited number of markers, which in some cases do not lead to the correct classification of samples. In any case, there is a general consensus on the fact that important metabolic changes are produced in the context of ccRCC.^16,20,24^

The use of LIMS to classify tumor samples has the advantage of discriminating between tumoral and nontumoral areas of a given section. The spatial resolution offered by this technique (10 μm/pixel or higher)^19,26^ permits segregation of necrotic areas or infiltrating cells, among others, and identification of different fingerprints in a given section, which may correspond to cell populations with different proliferation potential.

The combination of the powerful HPLC chromatographic separation and the sensitivity and resolving power of mass spectrometry makes HPLC-MS a technique of great identification potential. However, it requires larger samples and gives a single lipid fingerprint for each of them. In the case of tumors, this is an important drawback due to the highly heterogenic nature of the samples.^27^

The comparison between the results obtained from both techniques may serve as a cross-validation of the biomarkers obtained and to evaluate their individual performance. In addition, it is well-known that the analyte profile generated by electrospray ionization (ESI) and MALDI from the same sample are different. Comparison between both sets of data will allow us to evaluate how important such differences are for sample classification.

## Materials and Methods

### Materials and Reagents

1,5-Diaminonaphtalene (DAN), hematoxylin, eosin, ethanol (99.99% purity), HCl, toluene (analytical standard), ammonium formate (99.999%), and xylene for histological staining were purchased from Sigma-Aldrich (Steinheim, Germany). Water (Optima quality), acetonitrile, methanol, 2-propanol, and formic acid were purchased from Fisher Scientific (Fair Lawn, NJ). Chloroform and methanol for lipid extraction were from Scharlau (Barcelona, Spain)

### Kidney Sample Collection

The present study complies with current Spanish and European Union legal regulations. The samples were provided by the Basque Biobank for Research-OEHUN (www.biobancovasco.org). Each patient signed a specific document that had been approved by the Ethical and Scientific Committees of the Basque Country Public Health System (Osakidetza; PI+CES-BIOEF 2020-12).

Tumors and adjacent uninvolved kidney tissues from 40 ccRCC patients nephrectomized at Basurto and Galdakao-Usansolo University Hospitals were collected for the study. American Joint Committee on Cancer (AJCC, 2010)^28^ and WHO/ISUP’s criteria^29^ were used to assign the stage and grade, respectively. Table S1 describes the clinical and pathological characteristics of each individual case. Briefly, the series consisted of 14 females and 26 males, with a mean age of 64 years (from 37 to 83 years). Regarding the histological grade, 14 tumors were grade 2, 22 were grade 3, and 4 were grade 4. Regarding the tumor stage, 25 cases were stage I, 5 were stage II, 8 were stage III, and 2 were stage IV.

From each removed kidney, adjacent uninvolved tissue was separated from the ccRCC portion. Aliquots of each nontumoral and ccRCC tissue were stored fresh-frozen (−80 °C) in liquid nitrogen until extraction was performed. The remaining part was used to obtain serial sections of 15 μm for MALDI-IMS analysis. In this way, sections and tissue from uninvolved and ccRCC samples belonging to the same patients were analyzed by MALDI-IMS and HPLC-MS.

### HPLC-MS Experiments

Lipid extraction from tissue homogenates was performed by the Bligh and Dyer method,^30^ and HPLC-MS lipidomic analysis was done as described elsewhere.^31^ Briefly, each of the 80 samples was homogenized in 10 volumes of phosphate-buffered saline (10 mM phosphate buffer, pH 7.4, 150 mM NaCl) using a Polytron homogenizer (Kinematica AG, Malters, Switzerland): 12 mm dispersing aggregate, 1 min at 80% of maximum intensity, in an ice bath. Lipid extracts from human renal tissue were injected into an HPLC column coupled to a QExactive HF-X (Thermo Fisher) mass spectrometer. The analysis was performed in positive- and negative-ion modes, and the parameters were optimized using the Splash LipidoMix (Avanti Polar Lipids, Alabaster, AL) standard mixture. MS data were acquired and processed using the Xcalibur 4.1 package, with 5 ppm tolerance for lipid precursor and fragment ions.

LipidSearch 4.2.27 software (Mitsui Knowledge Industry, Tokyo, Japan) was used to identify and quantify the lipid species from classes: phosphatidylcholine (PC), phosphatidylethanolamine (PE), phosphatidylglycerol (PG), phosphatidylinositol (PI), phosphatidylserine (PS), lysophosphatidylcholine (LPC), phosphatidylcholine(ether/plasmalogen) (PC(O/P)), lysophosphatidylethanolamine (LPE), phosphatidylethanolamine(ether/plasmalogen) (PE(O/P)), lysophosphatidylglycerol (LPG), lysophosphatidylinositol (LPI), lysophosphatidylserine (LPS), sphingomyelin (SM), ceramide (Cer), monohexosylceramide (Hex1Cer), dihexosylceramide (Hex2Cer), sphingosine (SPH), sulfatide (SFT), cardiolipin (CL), diacylglycerol (DG), triacylglycerol (TG), cholesteryl ester (ChE), acylcarnitine (AcCa), and fatty acids (FA) were considered. Quantification was conducted via normalization of the intensity of the monoisotopic peak of each native species with respect to the intensity of the monoisotopic peak of the internal standard from Splash LipidoMix, Cer/Sph mixture I, Cardiolipin Mix I, 24:0(d4) l-carnitine, D18:1/12:0 monosulfogalactosyl (β) ceramide (NH_4_ salt), and oleic acid (d9) (Avanti Polar Lipids, Inc.) included in the lipid extraction step (Table S2).

The protein concentration of tissue homogenates was determined by the bicinchoninic acid method (Thermo Fisher Scientific) including 2% sodium dodecyl sulfate in all samples to avoid erroneous measures due to the presence of lipids.

For the statistical analysis, SPSS Statistics 17.0 (IBM, Armonk, NY) and Orange Biolab (Ljubljana, Slovenia) software were used.

### MALDI-IMS Experiments

Histological sections were prepared and analyzed by MALDI-IMS as described by Garate et al.^26^ Briefly, DAN was used as a matrix for negative-ion detection and deposited with the aid of our in-house designed sublimator.^32^ Sections were scanned in negative-ion mode at 10 μm/pixel for nontumoral tissue and 25 μm/pixel for ccRCC sections, using an orbitrap analyzer (MALDI-LTQ-Orbitrap XL, Thermo Fisher, San Jose, CA), equipped with a modified MALDI source.^33^

Data were acquired with a mass resolution of 60 000 at *m*/*z* = 400 and a laser energy of ∼10–20 μJ/pulse, depending on the spatial resolution. Two microscans of 10 laser shots were recorded for each pixel. Staining with H&E (Sigma-Aldrich Química, Madrid, Spain) was carried out for all biopsies once MALDI-IMS experiments were completed and the matrix was removed.

Spectra were processed using in-house developed software, built-in Matlab (MathWorks, Natick). Lipid assignment was achieved using the *m*/*z* value, the “on-tissue” MS/MS and MS^3^ data, and HPLC-ESI-MS/MS results.

Regarding lipid abundance, the MALDI-IMS protocol used in this work only gives relative abundances within each lipid class, because signal intensity cannot be translated directly into lipid abundance. Effects such as ion suppression or the different detectability of each lipid family preclude to establish a quantitative correlation between signal intensity and the relative abundance of lipid families, as it will be demonstrated by the comparison with the HPLC-MS data. Therefore, the discussion is limited to analyzing relative variations in the abundance of the species in a given family.

Data from each section were analyzed using a segmentation algorithm (HD-RCA)^34^ to isolate and identify the lipid signatures of each histological area in the section.

IBM SPSS Statistics for Windows (version 23.0; IBM, Chicago, IL) was used for Levene’s test and *T*-test performance. Levene’s test determines the homogeneity of the variables (H0 = groups have equivalent variances) to apply, if necessary, the appropriate correction. Orange (Bioinformatics Lab, University of Ljubljana, Slovenia) was used for principal component analysis (PCA) and for the development of classification models with different algorithms.

## Results and Discussion

### Imaging Lipid Distribution in Kidney Sections

In a previous publication, we demonstrated that using LIMS at 10 μm/pixel, it is possible to distinguish up to seven segments of the nephron and that the lipid fingerprint of each of the segments is conserved among individuals.^12^ Here, we have reduced spatial resolution to 25 μm/pixel to cover larger areas of tumor sections.

**Figure 1:**
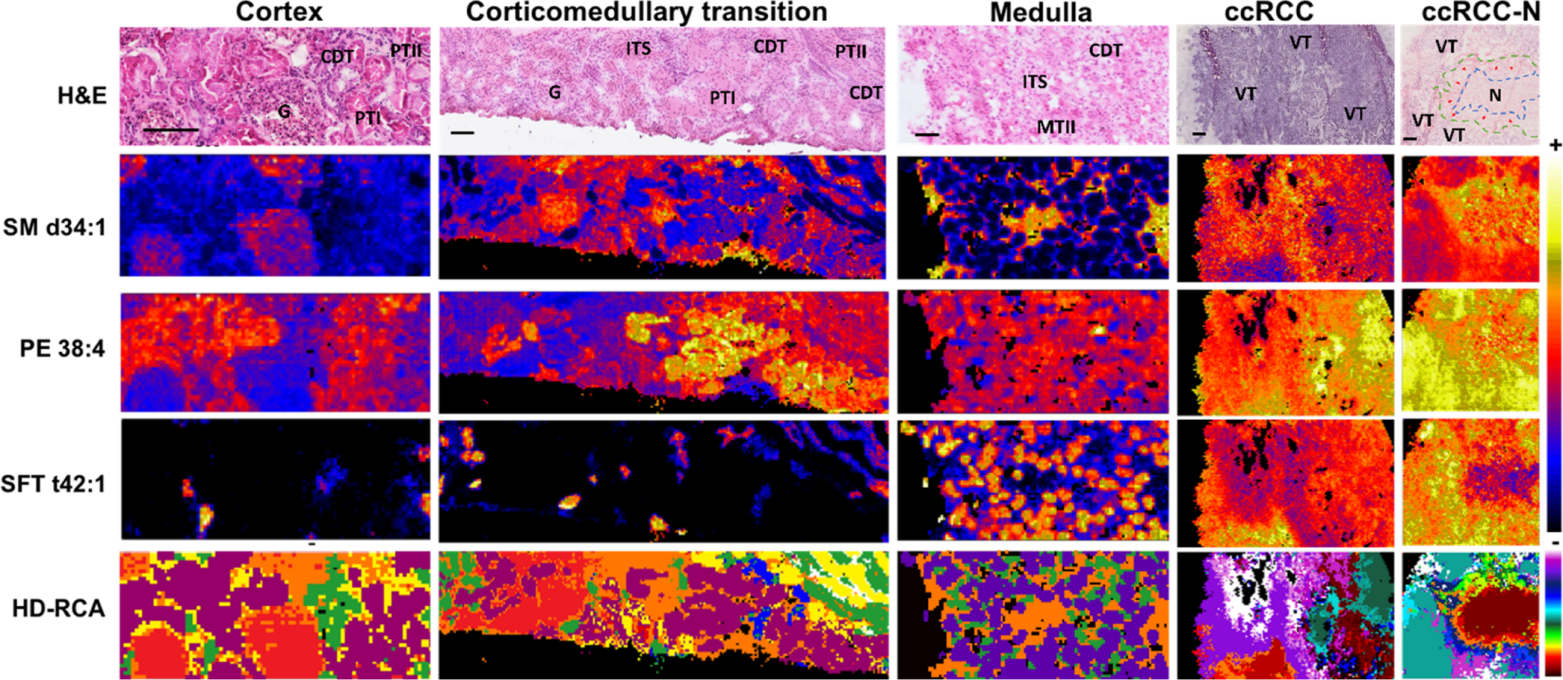
Comparison between the hematoxylin and eosin (H&E) microscopic image, the distribution of some example lipids, and the segmentation image for three sections of the healthy human kidney and two sections of ccRCC. Up to eight different lipid fingerprints were found in the normal/uninvolved part of the kidney, as previously reported.^12^ G: glomerulus; CMT: corticomedullary tubules; PTI: proximal tubules I; MTI: medullary tubules I; PTII: proximal tubules II; MTII: medullary tubules II; ITS: interstitial vascular structures; VT: tumor cells of a viable tumor showing a heterogeneous lipid fingerprint; N: tumor necrotic areas; red arrows: ischemic areas. An enlarged version of the necrotic areas may be found in Figure S1 in the Supporting Information (SI), together with an additional example. Scale bar = 150 μm.

**Figure 2:**
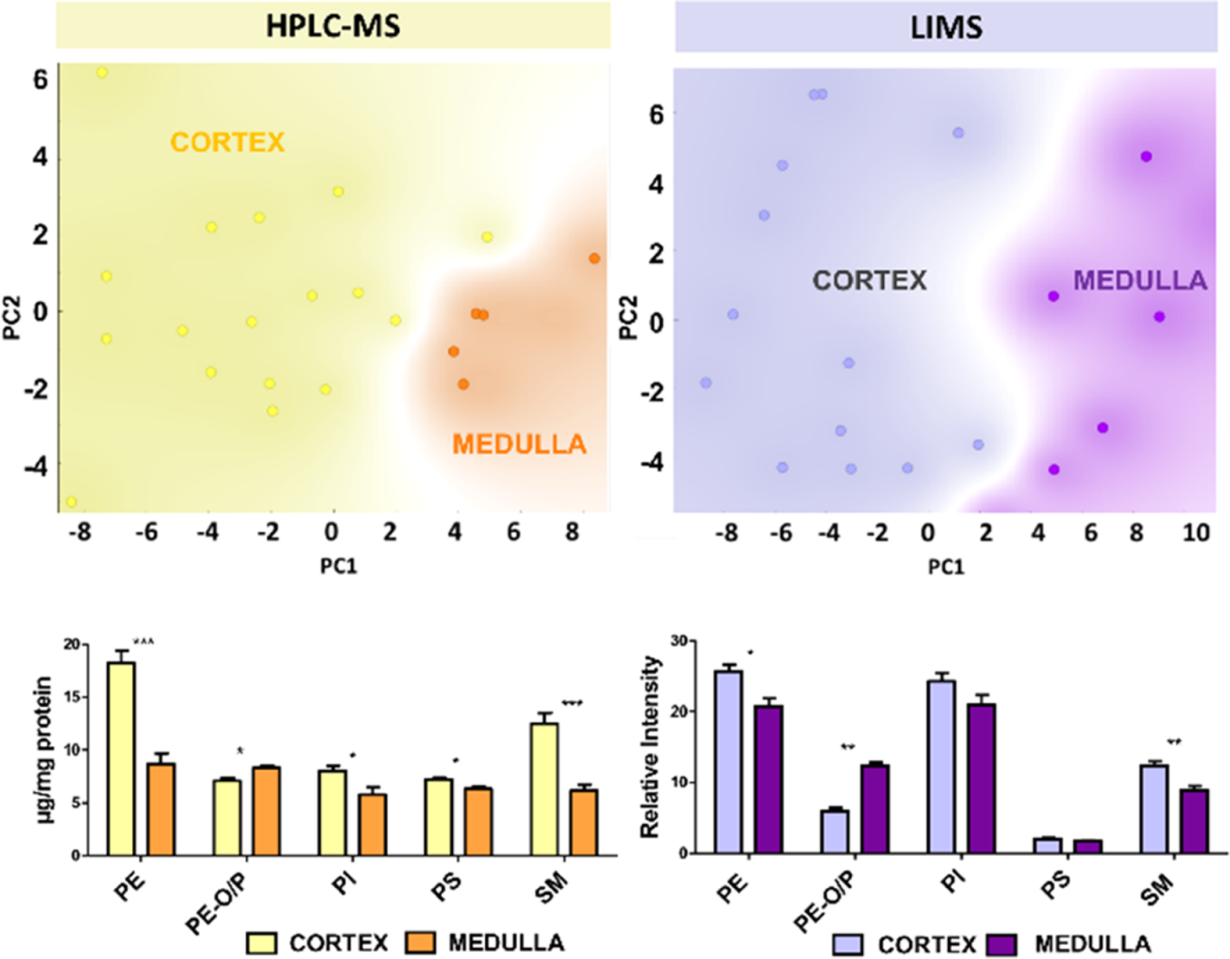
Scores plots of a PCA analysis of lipid fingerprints of kidney samples analyzed by HPLC-MS and LIMS and the relative abundance of the main lipid classes. Both sets of results were obtained in negative-ion mode. Only those species detected by both methods were considered. Therefore, sulfatides were not included, due to their low detectability in HPLC-MS with the protocol used.

Figure 1 shows some examples of lipid distribution across three sections of the uninvolved human kidney and two ccRCC sections. The kidney is a well-structured tissue. Its functional unit is the nephron: it starts in the glomerulus and projects a tubule that penetrates into the transition zone to surface back to the cortex and joins a collector tubule. The tubules are the main components of the medullary tissue and end in the renal pelvis.^35^ Thus, there is a clear difference in lipid composition between the renal cortex and the medulla (Figure 2). For example, the existence of a gradient in the relative abundance of sulfatides from the cortex to the medulla has been described.^36^

Conversely, ccRCC is a highly heterogeneous and disorganized tissue.^37,38^ Several cell populations with a variety of phenotypes are usually competing for resources and are found intermixed with necrotic and hypoxic areas. In this context, using an imaging technique to extract the lipid fingerprint of a tumor sample presents the advantage of enabling segregation of lipid signatures that do not correspond to tumor cells. The right column of Figure 1 shows the example of a tissue with a necrotic area that presents a characteristic and distinguishable lipid profile.

### Comparison between Lipid Fingerprints Obtained by LIMS and HPLC-MS

The chromatographic separation offered by HPLC has the clear advantage of minimizing ion suppression and overlapping between isobaric species. Furthermore, it is possible to include internal standards to perform relative quantification to a standard belonging to the same lipid class. However, it lacks the spatial information offered by LIMS. Substantially larger samples are required for HPLC-MS, and the whole tumor fragment provided by the pathologists is processed during lipid extraction. Thus, in principle, it is not clear if both techniques would yield similar results in the analysis of the same set of samples. To test this extent, we examined the complete set of samples using both techniques: LIMS and HPLC-MS. Figure 2 shows the comparison between the PCA analysis of the lipid fingerprints of the nontumor samples, while the results from three classification models may be found in the Supporting Information (Figure S2).

It is clear from Figure 2 that the cortex and the medulla present well-defined lipid fingerprints, and PCA analysis offers a good separation between both sets of samples. Careful examination of changes in lipid classes between the cortex and the medulla highlights the different detectability of lipid classes. For example, the PI class presents a strong signal in negative-ion mode, and therefore, its contribution to the LIMS spectrum is bigger than its relative abundance in the tissue, as highlighted by the quantitative data obtained by HPLC-MS. The opposite behavior was observed for PS, whose contribution to the LIMS overall spectrum is weak compared to its relative abundance.

Interestingly, changes in lipid classes follow the same trend in data obtained by both techniques: a decrease in PE, PS, and SM from the cortex to the medulla and an increase in PE-ether, although the magnitude of the changes is substantially different in some of the classes. It is worth mentioning that an important lipid class, sulfatides, was excluded from the comparison in Figure 2, as a very limited number of species were detected by HPLC-MS using our protocol.

The number of species detected and identified by each technique in negative-ion mode was very different: 148 by LIMS and 344 by HPLC-MS. Only those species detected by both methods were included in the comparison (Figure S3). Again, there is a good agreement in the changing trend between the cortex and the medulla, although the relative intensities of some species are strikingly different. The biggest differences were found in PE and PE-ether, probably due to the interference of isobaric PC species, which are detected as PC–CH_3_ in the imaging experiments.

### Characterization of the Lipidome of ccRCC

The comparison between results obtained with both techniques is not straightforward in the case of tumor samples, due to their high heterogeneity. Although the same samples were analyzed by both methods, the portion used for each technique may contain different cell populations. To circumvent this problem, 80 samples (40 uninvolved and 40 tumoral) were measured. Another issue to consider is that the spatial resolution offered by LIMS allowed us to isolate lipid signatures corresponding to tumor cell populations, while the lipid signature of the whole sample was obtained by HPLC-MS. Besides, no information regarding the nature of the sample was supplied. Therefore, classification of samples analyzed by HPLC-MS into the cortex/medulla was based on the information obtained with LIMS.

**Figure 3:**
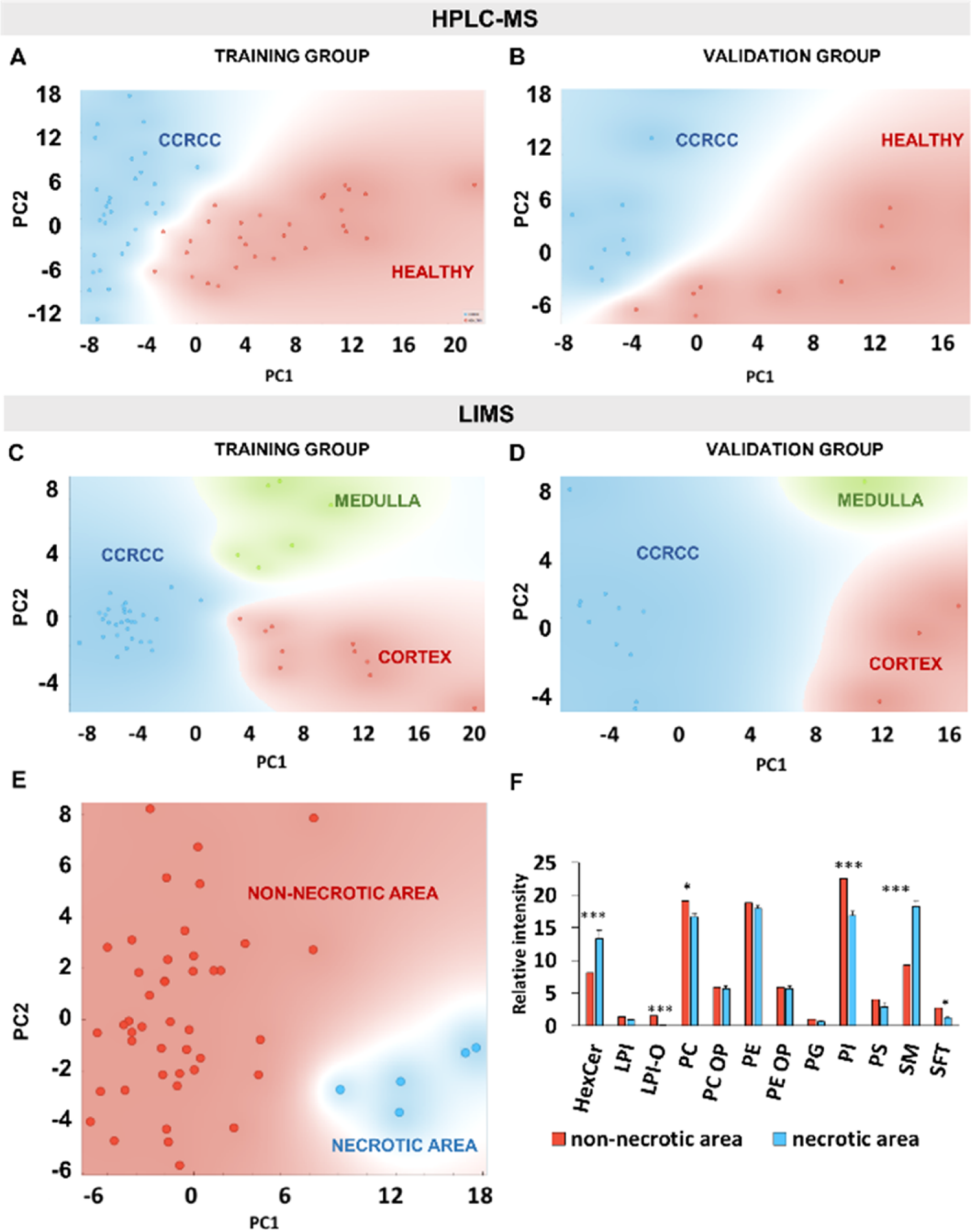
Scores plots of a PCA analysis of lipid signatures of healthy and tumor samples obtained using HPLC-MS (A, B) and LIMS (C, D). The spatial resolution of LIMS enabled selective extraction of the lipid signature of the necrotic areas (E), which is substantially different from those of the tumor cells (F). Red dots: healthy samples (A, B), healthy cortex samples (C, D), or non-necrotic lipid signatures in tumor samples (E); blue dots: tumor samples (A–D) or lipid signatures of necrotic areas (E).

Despite all these variables, comparable results were obtained by both techniques. Figure 3 shows PCA analyses of lipid fingerprints obtained using HPLC-MS (A, B) and LIMS (C, D). Samples were divided into training and validation sets: a panel of lipid biomarkers was extracted from the training set. Then, the validation group was analyzed using only that panel. A neat separation was obtained for both data sets. Among the three classification models tested (support vector machine (SVM), random forest, and logistic regression; Figure S4), the latter achieved an almost perfect classification of HPLC-MS samples in the training set, with only one misclassified sample, and a perfect classification in the validation group.

Regarding data obtained by LIMS, it was possible to classify samples into tumor, healthy cortex, and healthy medulla. Among the classification models (Figure S4), random forest correctly classified all samples both in training and validation sets. It seems that extracting the lipid fingerprint of the tumor cell populations had a (modest) beneficial effect on the classification models. The advantage of the spatial resolution also allowed us to identify and extract the lipid signature of necrotic areas (Figures 1, 3E, and S1), a histopathological sign of bad prognosis in ccRCC,^37^ which is also substantially different from that of tumor cells. Interpretation of other sources of heterogeneity of the lipid fingerprint in each section is a cumbersome task. ccRCC is a paradigmatic example of intratumor heterogeneity. Tumor cell populations that are apparently similar in an H&E section may have significant genomic and proteomic differences.^39^ In this study, zones of viable tumor that were described as similar by pathologists showed a highly heterogeneous lipid profile (see Figure 1), which suggests the existence of different tumor cell subpopulations from a metabolic point of view. Identification of these different cell subpopulations will need integrated studies of spatial genomics, proteomics, and lipidomics in histological sections of large areas of a significant number of ccRCCs, which is beyond the scope of the present work.

**Figure 4:**
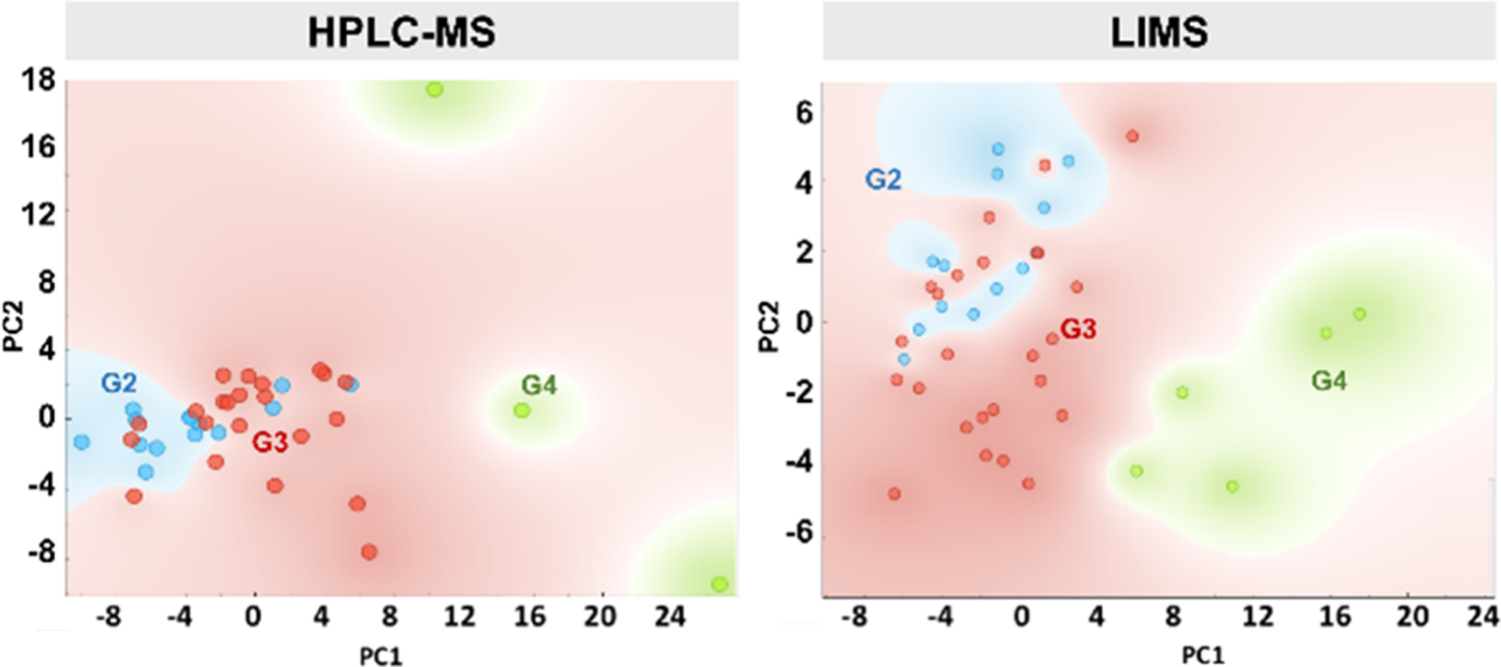
Scores plots of the PCA analysis of the lipid signatures of the tumor samples explored, identified according to their tumor grade. Left: lipid signature obtained by HPLC-MS; right: obtained by LIMS.

An attempt to correlate the histological grade (ISUP)^29^ with the lipid fingerprint was carried out (Figure 4). Clearly, the highest grade tumors (G4) showed a differential lipid profile compared to G2 and G3 tumors, whose lipid profiles appeared intermixed in the PCA analysis. Relative expression of PSs and sphingolipids such as SMs and HexCers was significantly higher in G4 tumors when compared to lower histological grade ones. This result agrees with the recent study of Młynarczyk et al.,^40^ which demonstrated increases in sphingolipid levels in high-grade ccRCCs. Recent works have also described that changes in the ceramide/SM balance and an increase in PSs in the outer leaflet of the cytoplasmic membrane are associated with the multidrug resistance (MDR) of cancer cells.^41,42^ Taken together, these results support the hypothesis that the dysregulation of sphingolipids and PSs contributes to the progression of ccRCC.^40^

### Comparison between Detected Species by HPLC-MS and LIMS

**Figure 5:**
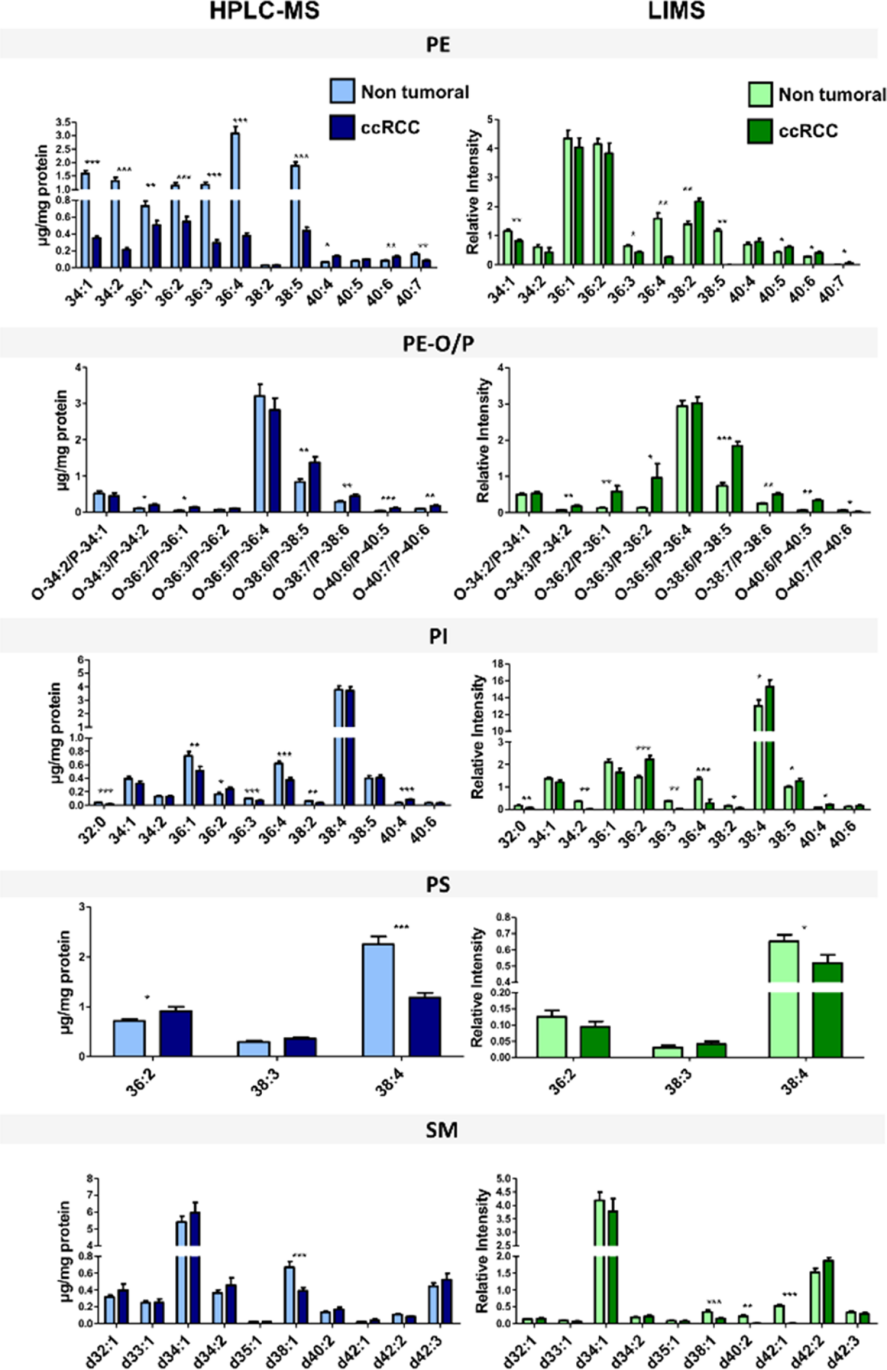
Lipid signatures of ccRCC and the uninvolved adjacent samples explored. Left panels: lipid signatures obtained by HPLC-MS; right panels: lipid signatures obtained by LIMS.

The final test to compare the performance of both techniques is to examine the changes in individual species. Figure 5 shows the comparison of the relative intensities of the lipid species, between nontumoral and tumoral samples. Only those species detected by both techniques were included, while the rest of the species may be found in the Supporting Information (Figures S6–S9). The concordance between both techniques is remarkable, taking into account the limitations described previously. Except for differences in relative intensities, the changes in most of the species follow the same trend in both data sets.

The main differences were found in PE species, which were very likely due to the interference of PC species. Nevertheless, both techniques report a generalized decrease in the abundance of individual PE species in tumor samples.

Regarding sphingomyelin (SM) species, LIMS reports a substantially larger relative abundance of SM d34:1 than HPLC-MS, although both techniques agree in labeling the changes in this species as not statistically significant. Perhaps the most striking difference in this class is the abundance of SM 42:2, which is over-represented in the LIMS data.

## Conclusions

We present a detailed study on the lipid signature of a healthy kidney and ccRCC, obtained by LIMS and HPLC-MS. Lipid profiles obtained by both techniques allowed us to classify kidney samples into the cortex and the medulla and to distinguish nontumoral from tumoral samples. Two main conclusions may be extracted from this observation. First, the limited number of species detected by LIMS is representative enough of the whole lipidome, even with the limitations of ionization suppression and overlapping of isobaric species. Second, spatial resolution is not a determinant factor to classify samples attending to their lipidome, at least in this case. The renal cortex and the medulla have very different compositions. The border between both areas is readily seen with the bare eye, and therefore, it is of no surprise that they present very different lipid fingerprints, as lipid composition is strongly connected to phenotype. A more precise classification of cell populations in each region certainly requires spatial resolution, either using LIMS or laser dissection prior to HPLC-MS analysis.

Regarding differences in lipid signature between uninvolved and ccRCC samples, the difference between both types of samples is so large that, once again, both the higher identification power of HPLC-MS and the image capabilities of LIMS enable obtaining a neat classification of samples. Nevertheless, the larger number of species identified in HPLC-MS is a clear advantage if one wants to extract biologically relevant conclusions regarding tumor transformation. Likewise, the ability of LIMS to distinguish different cell populations/phenotypes is of great interest to developing new protocols to automatically annotate samples based on their lipid profile. In this respect, the main conclusion may be that the overwhelming amount of data obtained by both samples is only a starting point for future investigations regarding the metabolic changes that originate the differences observed in lipid profiles.

## Supporting Information Available

Detailed view of two sections containing necrosis; performance of several classification models set up for classification of the samples; comparison between the change in individual species detected by both techniques; table with pathological parameters of all of the samples measured; and table with the species detected/quantified (PDF)
