# Supplementary material for: Using the Synergy between HPLC-MS and MALDI-MS Imaging to Explore the Lipidomics of Clear Cell Renal Cell Carcinoma
Source: Anal Chem. 2023 Jan 13;95(4):2285–93. doi: 10.1021/acs.analchem.2c03953 (PMC9893214; doi:10.1021/acs.analchem.2c03953)
Supplement: Supplementary file 1 — ac2c03953_si_001.pdf [file ac2c03953_si_001.pdf]

Supplemental Information for:

Using the Synergy Between HPLC-MS and MALDI-MS-Imaging to Explore the Lipidomics Of Clear Cell Renal Cell Carcinoma

Lucía Martín-Saiz[1], Beatriz Abad-García[2], Jon D. Solano-Iturri[3,4], Lorena Mosteiro[5], Javier Martín-Allende[1], Yuri Rueda[6], Amparo Pérez-Fernández[7], Miguel Unda[7], Pedro Coterón-Ochoa[8], Aintzane Goya[8], Alberto Saiz[9], Jennifer Martínez[9], Begoña Ochoa[6], Olatz Fresnedo[6], Gorka Larrinaga[4,10,11] and José A. Fernández[1*]

[1]Department of Physical Chemistry, Faculty of Science and Technology, University of the Basque Country (UPV/EHU), B. Sarriena, s/n, Leioa 48940, Spain.

[2]Central Analysis Service, Faculty of Science and Technology, University of the Basque Country (UPV/EHU), Leioa 48940, Spain.

[3]Service of Anatomic Pathology, Donostia University Hospital, Donostia/San Sebastian 20014, Spain.

[4]BioCruces Health Research Institute, Barakaldo 48903, Spain.

[5]Service of Anatomic Pathology, Cruces University Hospital, Barakaldo 48903, Spain.

[6]Lipids & Liver, Department of Physiology, Faculty of Medicine and Nursing, University of the Basque Country (UPV/EHU), B. Sarriena, s/n, Leioa 48940, Spain.

[7]Service of Urology, Basurto University Hospital, Bilbao 48003, Spain.

[8]Service of Urology, Galdakao-Usansolo University Hospital, Galdakao 48960,, Spain.

[9]Service of Anatomic Pathology, Galdakao-Usansolo University Hospital, Galdakao 48960, Spain.

[10]Department of Physiology, Faculty of Medicine and Nursing, University of the Basque Country (UPV/EHU), B. Sarriena, s/n, Leioa 48940, Spain.

[11]Department of Nursing I, Faculty of Medicine and Nursing, University of the Basque Country (UPV/EHU), B. Sarriena, s/n, Leioa 48940, Spain.

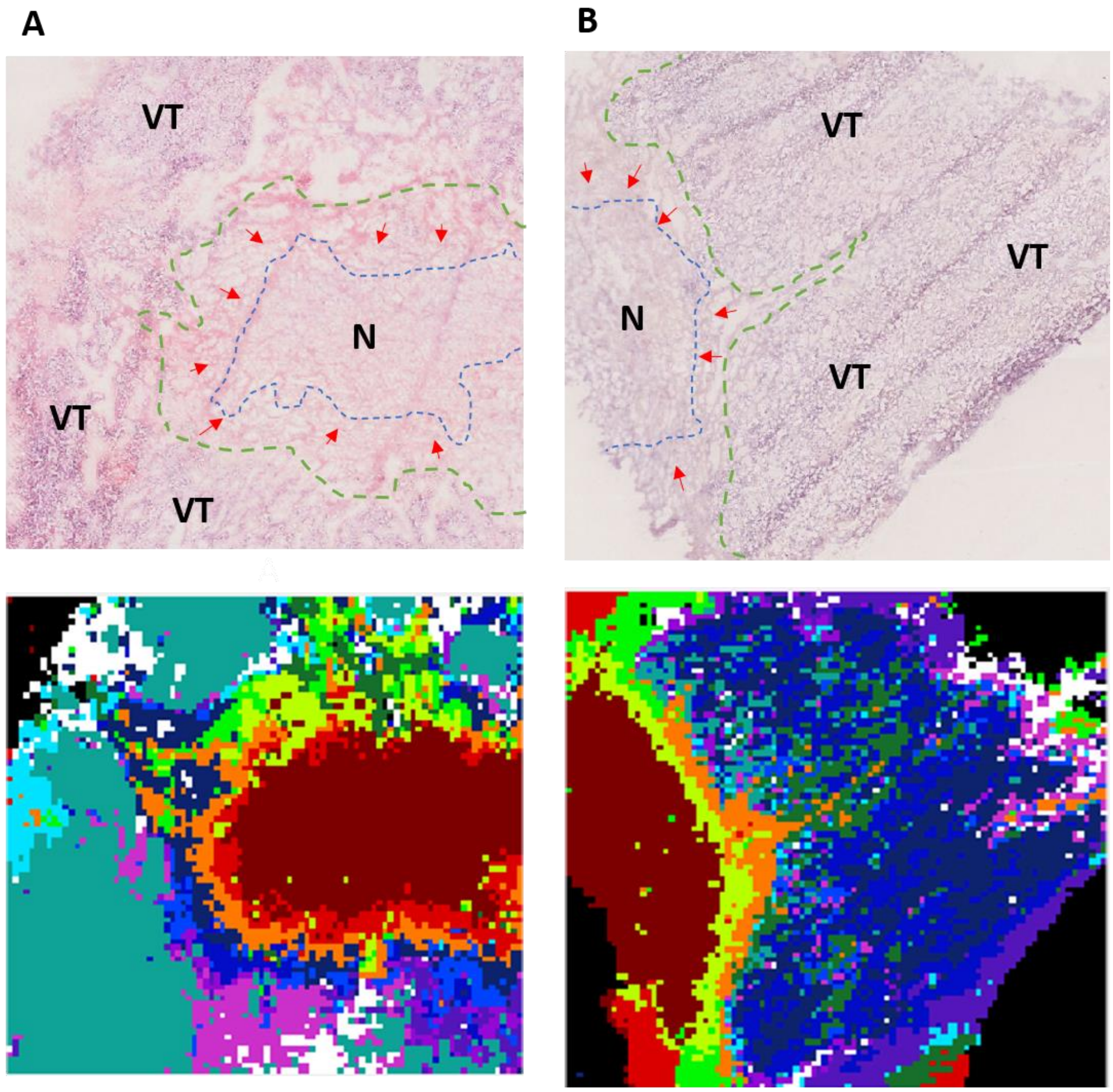

Figure S1. Comparison between the H&E microscopic image and the segmentation image for two sections of ccRCC containing necrotic areas. Images in A are enlarged versions of the ccRCC-N sample in **Figure 1.** Well-defined necrotic areas are surrounded by dotted lines in the H&E images. They seem to be ischemic necrosis areas due to preservation of non-ischemic peripheral neoplastic tubular pattern. Furthermore, LIMS images show that central and more developed ischemic areas (brown color) are surrounded by a fine orange rim, which correlates with also ischemic but recent and shadowy-preserved tubular structures. N=evolved ischemic necrosis; Red arrows=recent ischemic necrosis; VT= viable tumor portion. The clear alteration of the lipid profile in different degrees of ischemic tissue, something that it is difficult to detect in routine histology, points to the possible use of the lipid profile to classify ischemic samples in different points of evolution. Using the lipid profile, once associated to the different ischemic degrees, could help taking better advantage of small biopsies, in which only necrotic tissue is present, in order to carry out microdissections that, selecting ischemic portions of short evolution, may allow pathologists to apply molecular or immunohistochemistry studies, which are of high demand in the pathology of the XXI century.

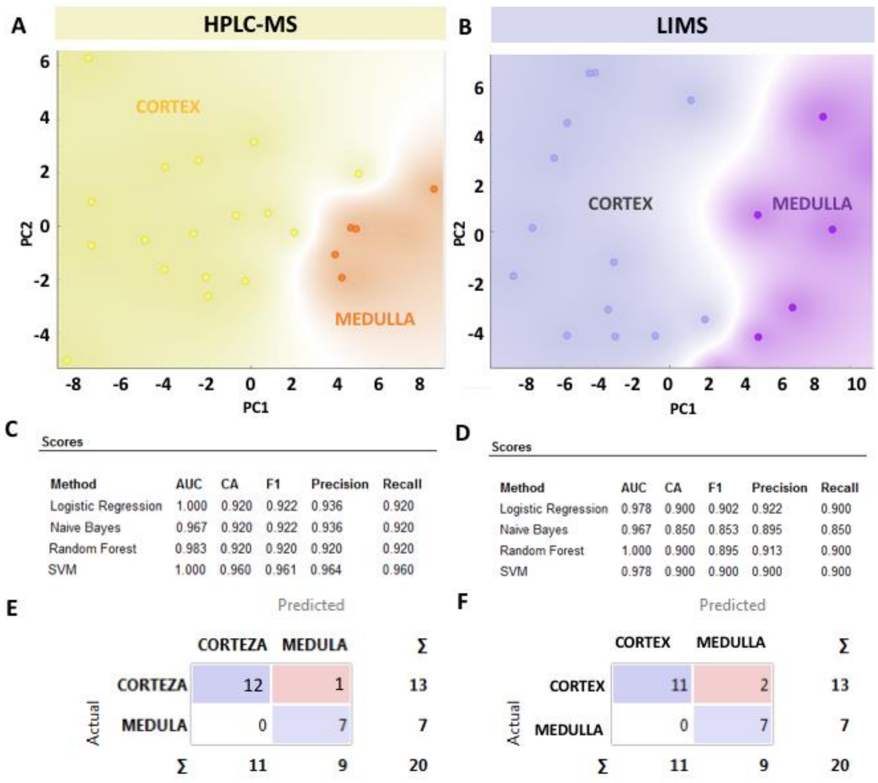

Method	AUC	CA	F1	Precision	Recall
Logistic Regression	1.000	0.920	0.922	0.936	0.920
Naive Bayes	0.967	0.920	0.922	0.936	0.920
Random Forest	0.983	0.920	0.920	0.920	0.920
SVM	1.000	0.960	0.961	0.964	0.960

Method	AUC	CA	F1	Precision	Recall
Logistic Regression	0.978	0.900	0.902	0.922	0.900
Naive Bayes	0.967	0.850	0.853	0.895	0.850
Random Forest	1.000	0.900	0.895	0.913	0.900
SVM	0.978	0.900	0.900	0.900	0.900

Actual \ Predicted	CORTEZA	MEDULA	Σ
CORTEZA	12	1	13
MEDULA	0	7	7
Σ	11	9	20

Actual \ Predicted	CORTEX	MEDULLA	Σ
CORTEX	11	2	13
MEDULLA	0	7	7
Σ	11	9	20

Figure S2. Lipid classification of healthy samples analyzed by HPLC-MS and LIMS. **(A,B)** Scores plot of the first two main components of the PCA model separating cortical and medullary samples analyzed by HPLC-MS and LIMS respectively. **(C,D)** Classification model's scores, **(E, F)** Matrix of confusion of Logistic Regression models.

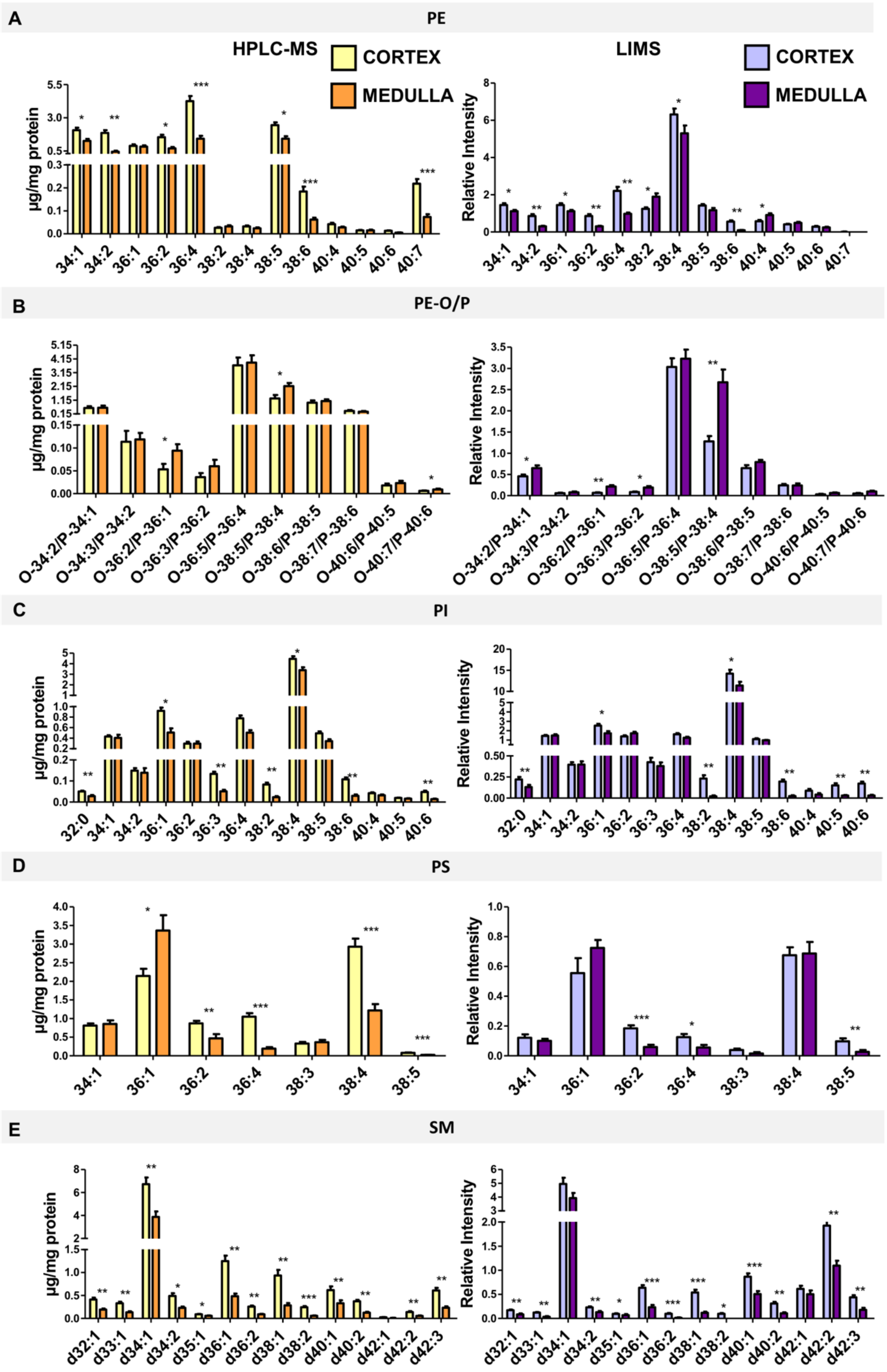

Figure S3. Comparison of individual glycerophospholipids and sphingolipids concentration and relative intensity between cortex and medulla samples studied by HPLC-MS and LIMS respectively. HPLC-MS values are expressed in µg/mg protein while LIMS data are expressed by relative intensity. Values are expressed as mean ± SEM (n=15 for cortical samples and n=5 for medulla samples). Statistical analysis was assessed using T-test analysis. * = p-value<0.05; ** = p-value<0.01; *** =p-value<0.001

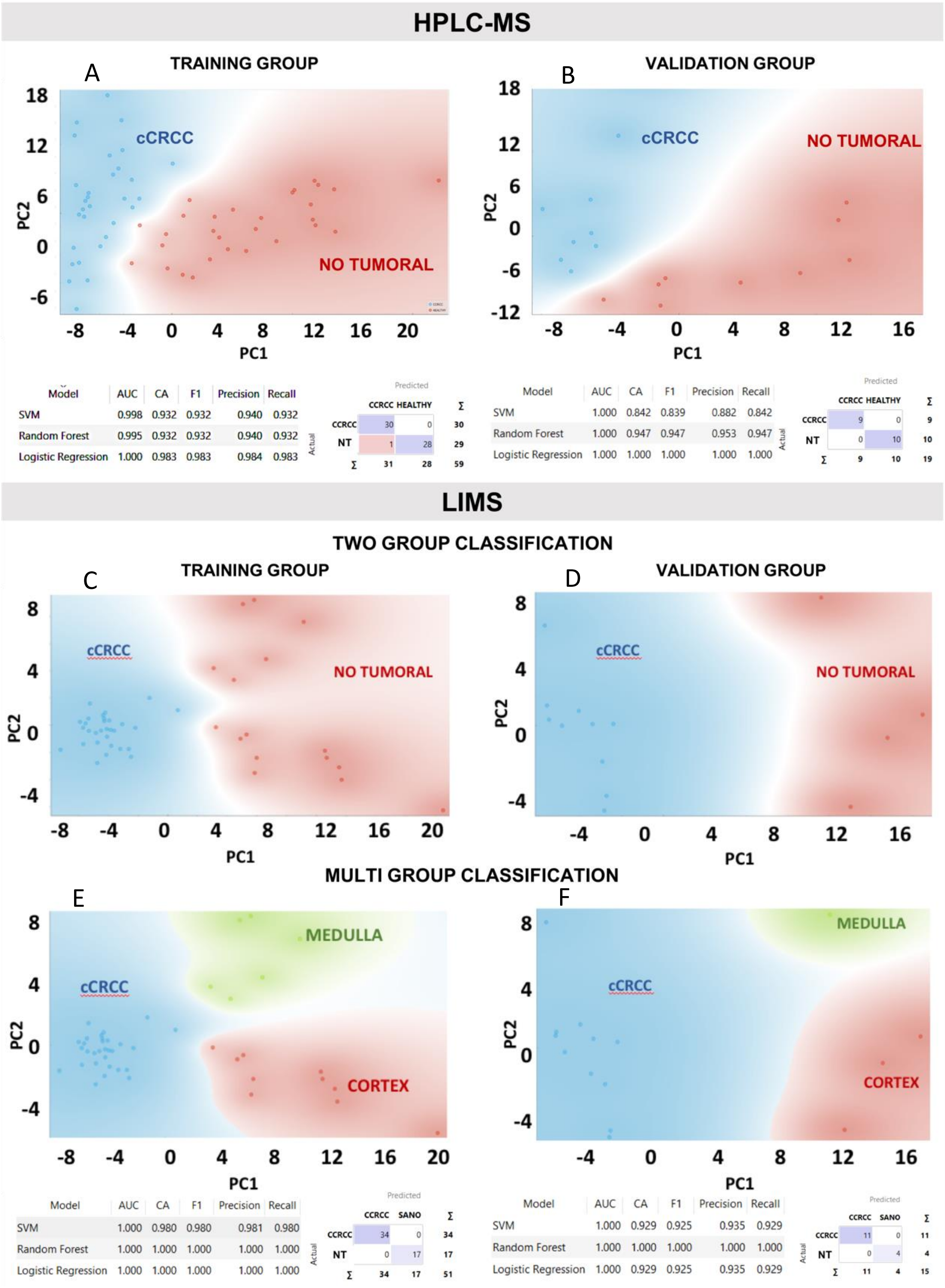

Figure S4. Lipid classification of non-tumoral and ccRCC samples analyzed by HPLC-MS and LIMS. Scores plot of the first two main components of the PCA model separating cortical and medullary samples **together with the performance of three classification models tested and the** confusion matrix of Logistic Regression for the data analyzed by HPLC-MS **(A, B),** and LIMS **(C-F)**.

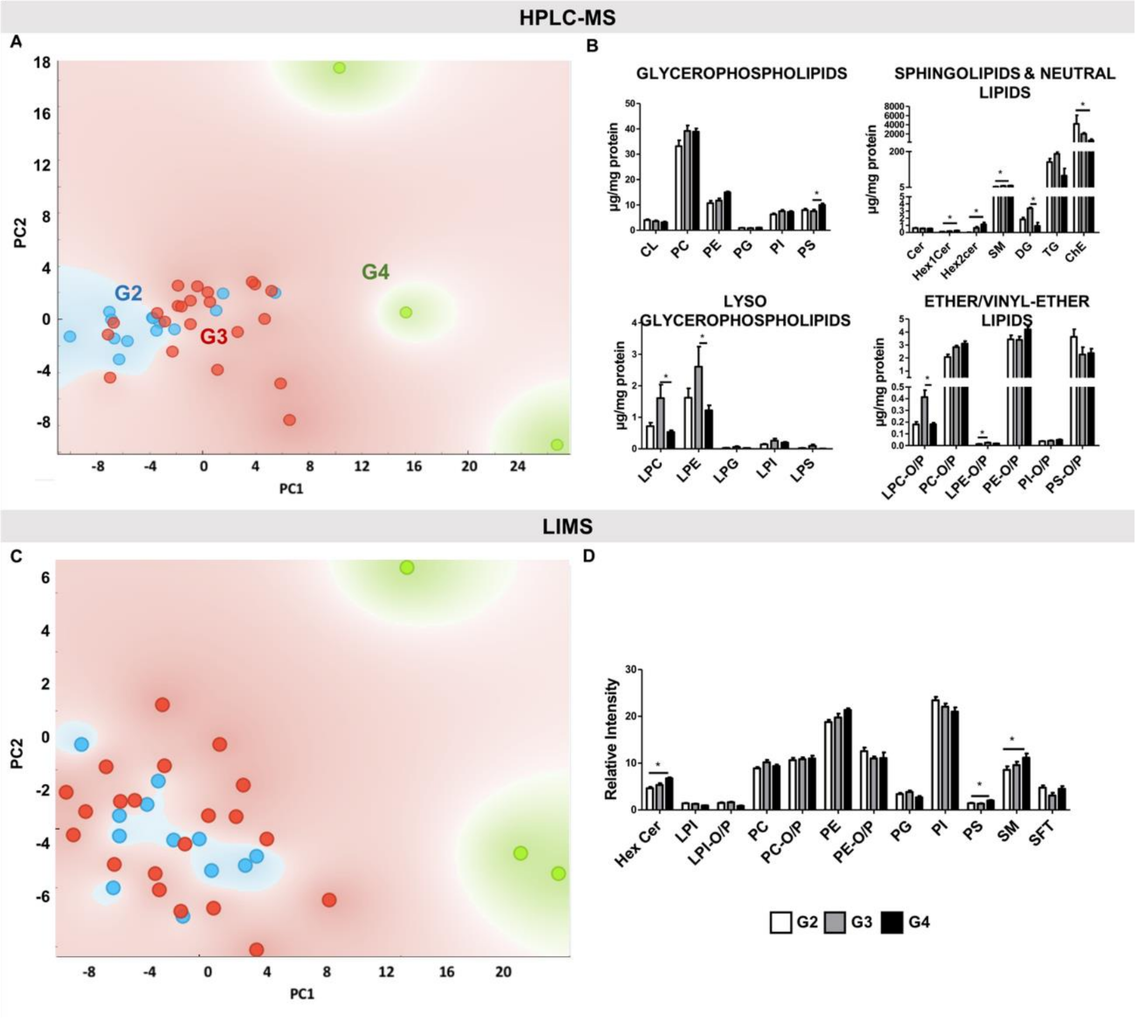

Figure S5. PCA analysis of the lipid signatures of the tumor samples explored, identified according to their tumor grade. (**A** and **B**) PCA and lipid signature obtained by HPLC-MS; (**C** and **D**) obtained by LIMS.

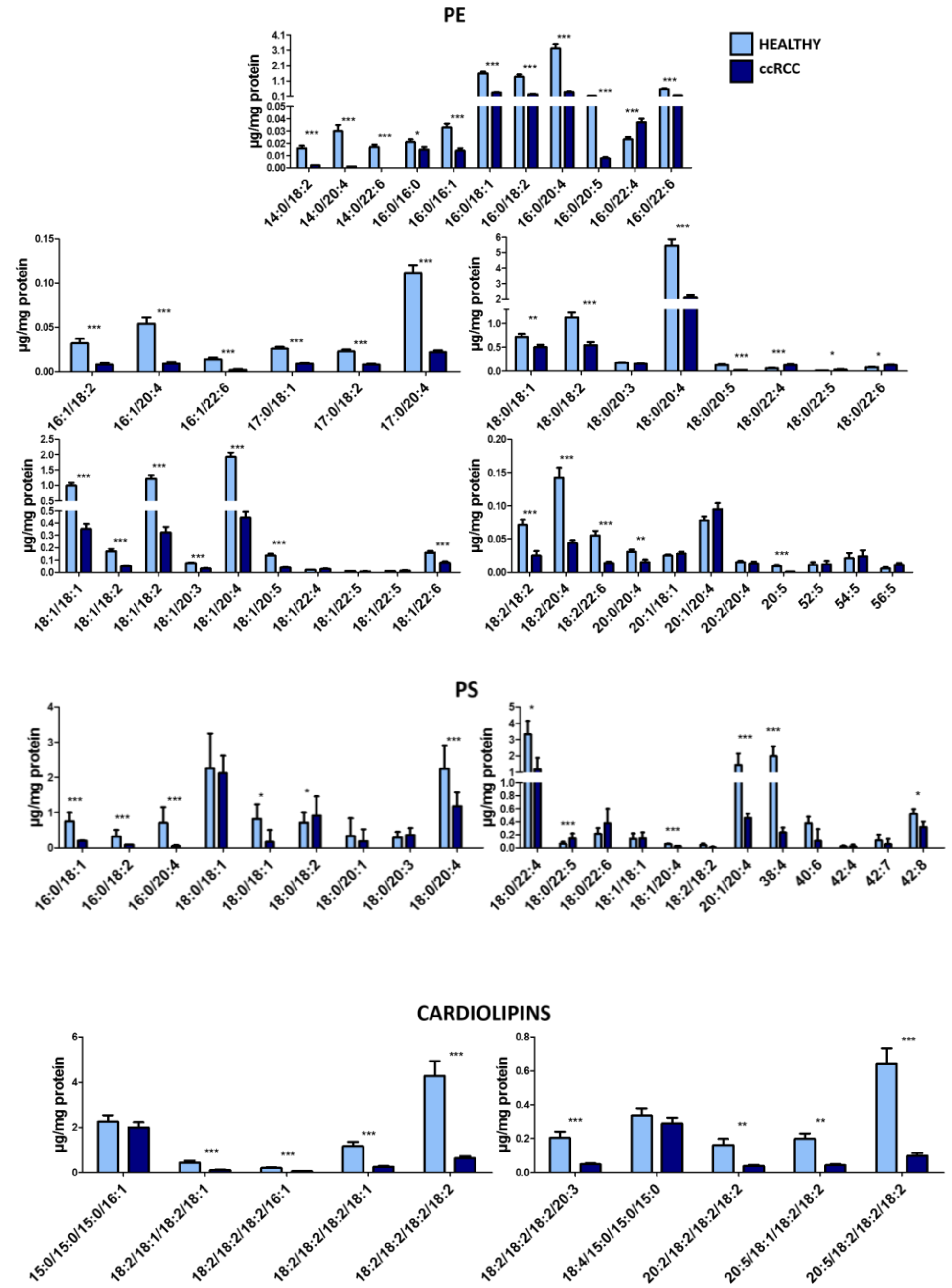

Figure S6. Comparison of the concentration of individual glycerophospholipids belonging to phosphatidylethanolamine (PE), cardiolipin (CL) and phosphatidylserine (PS) lipid classes between non tumoral and ccRCC renal samples detected by HPLC-MS. Values are expressed as mean ± SEM (n=40 for both groups). Statistical analysis was assessed using T-test analysis. * = p-value<0.05; ** = p-value<0.01; *** = p-value<0.001.

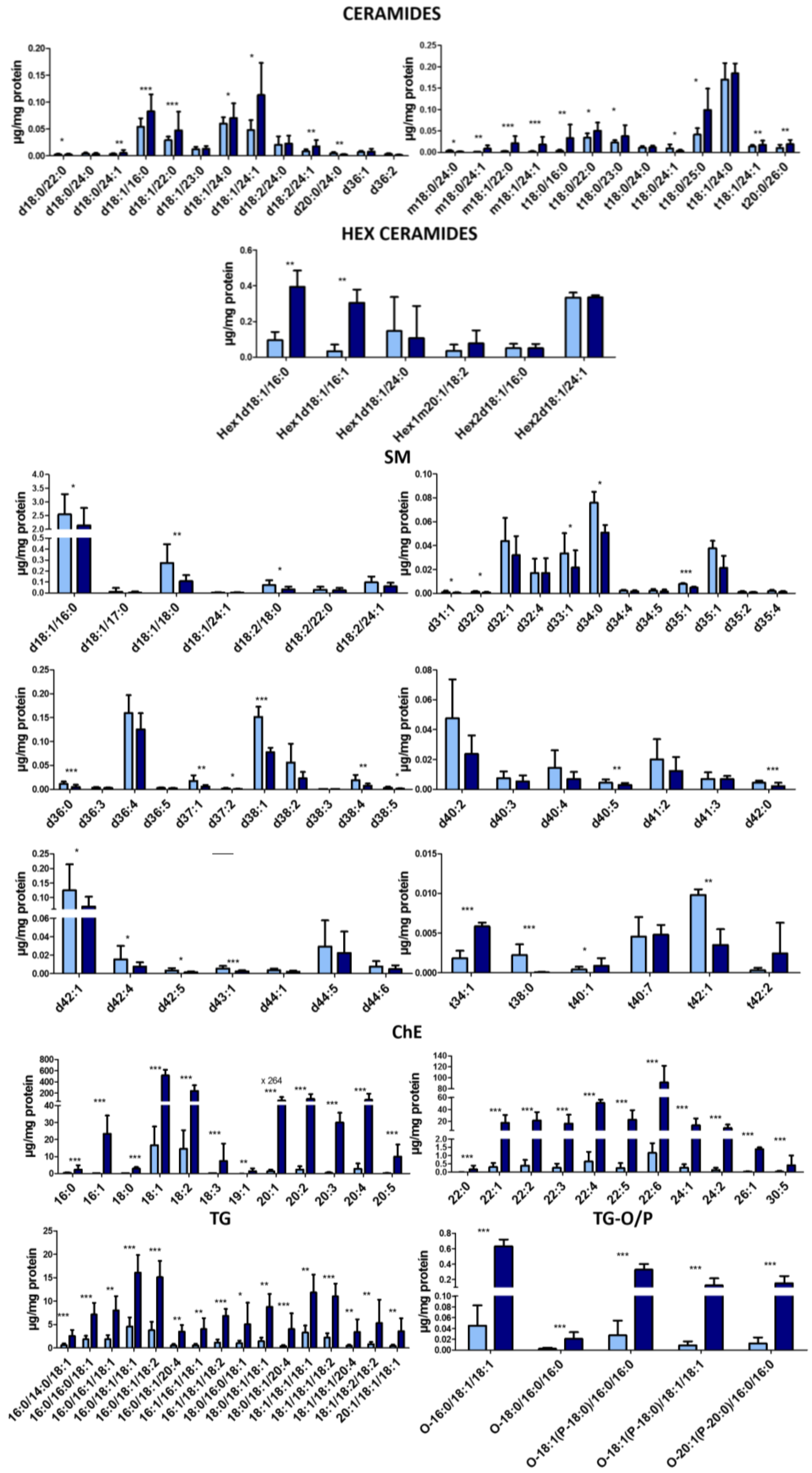

Figure S7. Comparison of the concentration of individual sphingolipids and neutral lipids belonging to ceramides (Cer), monohexosylceramide (Hex1Cer), dihexosylceramide (Hex2Cer), sphingomyelin (SM), cholesteryl ester (ChE), triacylglyceride (TG) and ether triacylglyceride lipid classes between non tumoral (light blue) and ccRCC (dark blue) renal samples detected by HPLC-MS. Values are expressed as mean ± SEM (n=40 for both groups). Statistical analysis was assessed using T-test analysis. * = p-value<0.05; ** = p-value<0.01; *** = p-value<0.001.

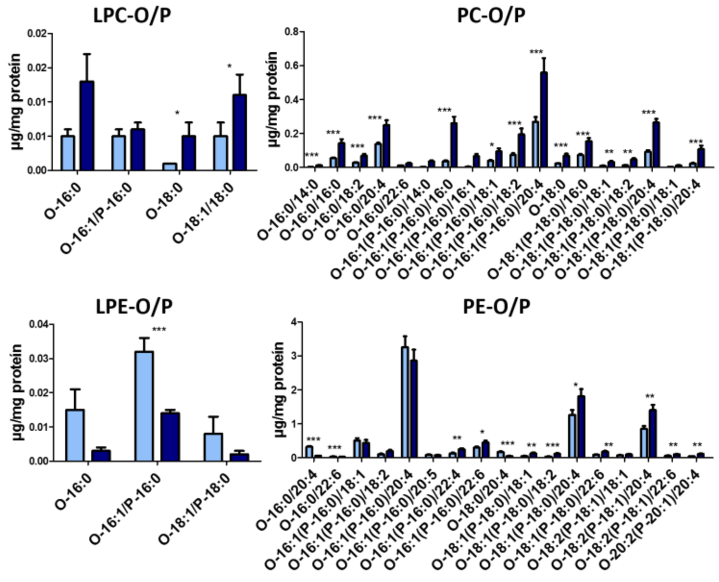

Figure S8. Comparison between the concentration of individual ether/vinyl ether glycerophospholipids belonging to ether/vinyl ether lysophosphatidylcholine (LPC-O/P), ether/vinyl ether phosphatidylcholine (PC-O/P), ether/vinyl ether lysophosphatidylethanolamine (LPE-O/P), ether/vinyl ether phosphatidylethanolamine (PE-O/P) lipid classes between non tumoral (light blue) and ccRCC (dark blue) renal samples detected by HPLC-MS. Values are expressed as mean ± SEM (n=40 for both groups). Statistical analysis was assessed using T-test analysis. * = p-value<0.05; ** = p-value<0.01; *** = p-value<0.001.

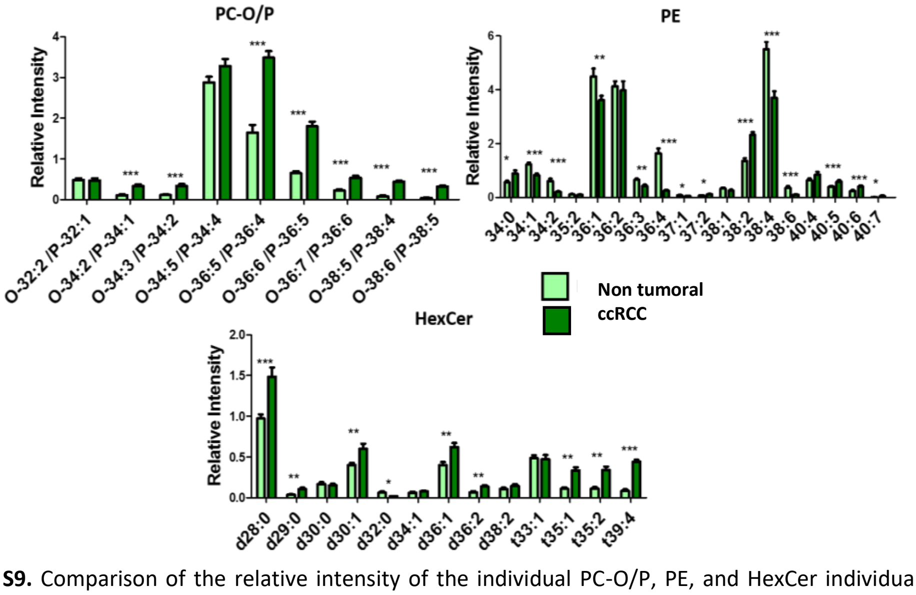

Figure S9. Comparison of the relative intensity of the individual PC-O/P, PE, and HexCer individual lipid species between non tumoral and ccRCC renal samples detected by LIMS. Values are expressed as mean ± SEM (n=20 for non-tumoral group and n=40 for ccRCC group). Statistical analysis was assessed using T-test analysis. * = p-value<0.05; ** = p-value<0.01; *** = p-value<0.001.

Table S1. Clinical and pathological parameters of ccRCC patients. American Joint Committee on Cancer (AJCC 2010) [1] and WHO/ISUP [2] criteria were applied to assign Stage and Grade, respectively.

Patient Number	Sex	Age	Histological Grade	TNM Stage
**1**	Female	60	2	I
**2**	Male	64	2	I
**3**	Female	60	3	II
**4**	Female	66	2	I
**5**	Male	58	2	I
**6**	Male	61	4	III
**7**	Male	77	3	I
**8**	Male	61	3	I
**9**	Male	74	2	I
**10**	Male	68	2	I
**11**	Male	62	3	I
**12**	Male	64	3	I
**13**	Male	52	3	I
**14**	Male	65	3	II
**15**	Male	56	2	I
**16**	Female	56	2	I
**17**	Male	66	3	III
**18**	Male	58	3	I
**19**	Female	57	2	IV
**20**	Female	62	3	I
**21**	Male	71	2	I
**22**	Male	66	3	III
**23**	Male	57	3	I
**24**	Female	64	3	III
**25**	Male	67	3	I
**26**	Female	81	2	I
**27**	Male	48	3	I
**28**	Male	52	4	IV
**29**	Female	77	2	II
**30**	Male	59	3	II
**31**	Female	56	2	I
**32**	Female	73	3	I
**33**	Male	56	3	I
**34**	Male	73	3	III
**35**	Male	83	3	III
**36**	Male	82	2	I
**37**	Female	47	3	II
**38**	Female	60	3	I
**39**	Female	71	4	III
**40**	Female	70	4	III

[1] Edge, S.B.; Byrd, D.R.; Compton, C.C.; Fritz, A.G.; Greene, F.L.; Trotti, A. *AJCC Cancer Staging Manual, 7th ed*.; Springer: Berlin/Heidelberg, Germany, **2010**.
[2] Delahunt, B.; Cheville, J. C.; Martignoni, G.; Humphrey, P. A.; Magi-Galluzzi, C.; McKenney, J.; Egevad, L.; Algaba, F.; Moch, H.; Grignon, D. J.; Montironi, R.; Srigley, J. R.; Members of the ISUP Renal Tumor Panel. The International Society of Urological Pathology (ISUP) grading system for renal cell carcinoma and other prognostic parameters *Am. J. Surg. Pathol.* **2013**, *37*, 1490-1504.

Table S2. Number of lipid species quantified by HPLC-MS.

Lipid Class	Polarity		Internal standard used for relative quantification				
	ESI+	ESI-	Internal Standard Mix	Compound Name	Exact Mass	Chemical Formule	Conc. (µg/mL)
PC	92	73	Splah Lipidomix Avanti Number: 330707 (Avanti Polar Lipids)	15:0-18:1(d7) PC	752.6055	C41H73D7NO8P	160.7
PE	69	73		15:0-18:1(d7) PE	710.5586	C38H67D7NO8P	5.7
PG	11	27		15:0-18:1(d7) PG (Na Salt)	763.5351	C39H67D7NaO10P	29.1
PI	25	30		15:0-18:1(d7) PI (NH4 Salt)	846.5958	C42H75D7NO13P	9.1
PS	18	24		15:0-18:1(d7) PS (Na Salt)	776.5304	C39H66D7NNaO10P	4.2
LPC	21	20		18:1(d7) Lyso PC	528.3915	C26H45D7NO7P	25.5
LPE	11	19		18:1(d7) Lyso PE	486.3446	C23H39D7NO7P	5.3
LPG	*n.d.	7					
LPI	1	8					
LPS	2	6					
SM	55	29		D18:1-18:1(d9) SM	737.6392	C41H72D9N2O6P	30.9
ChE	23	n.d.		18:1(d7) Chol Ester	657.6436	C45H71D7O2	356.1
TG	129	n.d.		15:0-18:1(d7)-15:0TAG	811.7641	C51H89D7O6	57.3
DG	9	n.d.		15:0-18:1(d7) DAG	587.5501	C36H61D7O5	9.4
Cer	27	12	Ceramide/Sphingoid Internal Standard Mixture I Avanti: LM-6002 (Avanti Polar Lipids)	Ceramide (C12)	481.4495	C30H59NO3	12.3
Hex1Cer	10	3		Glucosyl(β) C12 Ceramide	643.5023	C36H69NO8	16.5
Hex2Cer	4	2		Lactosyl(β) C12 Ceramide	805.5550	C42H79NO13	21.0
SPH	4	n.d.		Sphingosine (C17)	285.2668	C17H35NO2	7.2
ST	1	n.d.	Avanti 860573 (Avanti Polar Lipids)	D18:1/12:0 monosulfogalactosyl(β) ceramide (NH4 Salt)	740.4860	C36H72N2O11S	50.0
CL	8	10	Cardiolipin internal standard mixture Avanti LM6003 (Avanti Polar Lipids)	14:1(3)-15:1 CA (NH4 Salt)	1246.8001	C66H120O17P2	100.8
AcCa	18	n.d.	Avanti 870857P (Avanti Polar Lipids)	24:0(d4) L-carnitine	516.8400	C31H57D4NO4	47.6
FA	n.d.	6	Avanti 861809O (Avanti Polar Lipids)	Oleic acid (d9)	291.3120	C18H25D9O2	51.2

- Not detected

Table S3. Lipid molecules detected by HPLC-MS in the healthy and ccRCC samples.

Lipid Molecule	ccRCC/healthy (µg/mg protein ratio)	t-test	significance
Cer(d18:1_16:0)	1.86699	0.00000	***
Cer(d18:1_22:0)	1.60214	0.00047	***
Cer(d18:1_24:0)	1.28973	0.02058	*
Cer(d18:1_24:1)	2.56036	0.00000	***
Cer(d18:2_24:0)	1.23924	0.11657	
Cer(d32:1)	1.24712	0.03684	*
Cer(d41:1)	1.14015	0.18680	
Cer(d42:0)	1.24872	0.16829	
Cer(d42:1)	2.26785	0.00589	**
Cer(d42:3)	2.19916	0.00003	***
Cer(t17:0_25:0)	0.43218	0.00075	***
Cer(t20:0_26:0)	0.89654	0.66675	
CL(15:0_15:0_15:0_16:1)	0.89128	0.48406	
CL(18:2_18:1_18:2_18:1)	0.26545	0.00026	***
CL(18:2_18:2_18:2_16:1)	0.32683	0.00008	***
CL(18:2_18:2_18:2_18:1)	0.22430	0.00003	***
CL(18:2_18:2_18:2_18:2)	0.14866	0.00000	***
CL(18:2_18:2_18:2_20:3)	0.24050	0.00011	***
CL(18:4_15:0_15:0_15:0)	0.86829	0.39490	
CL(20:2_18:2_18:2_18:2)	0.23781	0.00104	**
CL(20:5_18:1_18:2_18:2)	0.22387	0.00002	***
CL(20:5_18:2_18:2_18:2)	0.15486	0.00000	***
Hex1Cer(d34:1)	2.99907	0.00024	***
Hex1Cer(d42:1)	2.01220	0.00093	***
Hex1Cer(d42:2)	3.85218	0.00001	***
Hex2Cer(d34:1)	14.14168	0.02811	*
Hex2Cer(d42:2)	6.52452	0.02878	*
LPC(14:0)	3.51905	0.03745	*
LPC(15:0)	0.76749	0.66595	
LPC(16:0)	0.87015	0.69009	
LPC(16:0e)	2.52449	0.05786	
LPC(16:1)	3.89545	0.03444	*
LPC(16:1e)	1.21694	0.31297	
LPC(17:0)	0.99419	0.98521	
LPC(18:0)	1.00833	0.97313	
LPC(18:0e)	4.09340	0.03484	*
LPC(18:1)	0.49290	0.33162	
LPC(18:1e)	2.13456	0.14247	
LPC(18:2)	1.00637	0.99173	
LPC(20:1)	1.65635	0.33685	
LPC(20:2)	1.67574	0.30830	
LPC(20:3)	1.08183	0.84522	
LPC(20:4)	1.41722	0.39400	

LPC(20:5)	1.06462	0.88560	
LPC(22:4)	3.43791	0.07560	
LPC(22:5)	1.86305	0.17822	
LPC(22:6)	1.09997	0.82051	
LPE(15:0)	0.30752	0.18381	
LPE(16:0)	0.24089	0.00154	**
LPE(16:0e)	0.20904	0.06345	
LPE(16:1)	1.42323	0.43633	
LPE(16:1e)	0.43241	0.00003	***
LPE(17:0)	0.14264	0.00071	***
LPE(18:0)	0.15582	0.00003	***
LPE(18:1)	0.48061	0.11403	
LPE(18:1e)	0.23481	0.24542	
LPE(18:2)	0.84283	0.71893	
LPE(20:1)	1.66658	0.32360	
LPE(20:2)	1.17842	0.72028	
LPE(20:3)	0.97148	0.94321	
LPE(20:4)	0.73437	0.39864	
LPE(20:5)	0.57017	0.17867	
LPE(22:3)	0.86454	0.73443	
LPE(22:4)	1.81735	0.15917	
LPE(22:5)	1.46448	0.36199	
LPE(22:6)	1.03219	0.93156	
LPG(14:1)	0.09814	0.26604	
LPG(15:0)	0.43184	0.22917	
LPG(16:0)	0.33850	0.02668	*
LPG(18:1)	1.68154	0.44187	
LPG(18:2)	0.80323	0.74819	
LPG(20:4)	0.91047	0.85580	
LPG(22:6)	0.26929	0.00383	**
LPI(16:0)	0.44461	0.04646	*
LPI(18:0)	0.55655	0.10170	
LPI(18:1)	0.82294	0.57900	
LPI(18:2)	0.96496	0.93268	
LPI(20:2)	0.33863	0.05646	
LPI(20:3)	0.50959	0.11813	
LPI(20:4)	0.65506	0.29999	
LPI(22:6)	0.44277	0.05968	
LPS(16:0)	0.19282	0.12775	
LPS(18:0)	0.42326	0.25019	
LPS(18:1)	0.35627	0.31164	
LPS(18:2)	0.79798	0.78288	
LPS(19:0)	0.16517	0.24156	
LPS(20:4)	0.31602	0.11182	
PC(14:0_14:0)	1.83405	0.02296	*
PC(14:0_18:2)	1.51048	0.04350	*

PC(14:0_20:4)	0.57619	0.00670	**
PC(14:0_22:6)	0.20301	0.00000	***
PC(15:0_14:0)	2.41500	0.02378	*
PC(15:0_16:0)	1.94348	0.00951	**
PC(15:0_16:1)	3.51539	0.00766	**
PC(15:0_18:1)	1.17877	0.29346	
PC(15:0_18:2)	0.94573	0.71158	
PC(15:0_20:4)	1.10311	0.51014	
PC(15:0_20:4)	0.60808	0.00580	**
PC(16:0_14:0)	1.35389	0.03744	*
PC(16:0_16:0)	1.48399	0.00081	***
PC(16:0_16:1)	2.13053	0.00005	***
PC(16:0_17:0)	1.64378	0.00619	**
PC(16:0_18:1)	0.66197	0.00003	***
PC(16:0_18:2)	0.86921	0.14591	
PC(16:0_18:3)	0.84706	0.20552	
PC(16:0_20:3)	0.69177	0.00072	***
PC(16:0_20:4)	0.65192	0.00003	***
PC(16:0_20:5)	0.52795	0.00083	***
PC(16:0_22:4)	1.77968	0.00009	***
PC(16:0_22:6)	0.50163	0.00000	***
PC(16:0e_14:0)	4.14974	0.00012	***
PC(16:0e_16:0)	2.66314	0.00038	***
PC(16:0e_18:2)	2.43201	0.00135	**
PC(16:0e_20:4)	1.81064	0.00038	***
PC(16:0e_22:6)	2.04307	0.01451	*
PC(16:1_14:0)	2.66654	0.00359	**
PC(16:1_18:2)	1.37914	0.05466	
PC(16:1_20:4)	0.89387	0.56684	
PC(16:1_22:6)	0.31917	0.00001	***
PC(16:1e_14:0)	14.47892	0.00000	***
PC(16:1e_16:0)	7.16946	0.00000	***
PC(16:1e_16:1)	14.72809	0.00001	***
PC(16:1e_18:1)	2.44489	0.02009	*
PC(16:1e_18:1)	2.44947	0.00231	**
PC(16:1e_18:2)	2.62108	0.00284	**
PC(16:1e_20:4)	2.09930	0.00242	**
PC(17:0_18:1)	0.54415	0.00002	***
PC(17:0_18:2)	1.05076	0.74106	
PC(17:0_18:2)	0.81889	0.11171	
PC(17:0_20:4)	0.86692	0.16765	
PC(17:1_18:2)	0.40419	0.05695	
PC(17:1_20:4)	0.29245	0.00657	**
PC(18:0_16:0)	1.66626	0.00175	**
PC(18:0_18:1)	0.96881	0.79571	
PC(18:0_18:2)	1.38504	0.00776	**

PC(18:0_20:3)	1.09918	0.43837	
PC(18:0_20:4)	1.30590	0.01069	*
PC(18:0_22:4)	2.68036	0.00000	***
PC(18:0_22:5)	0.87339	0.65998	
PC(18:0_22:6)	1.12367	0.38612	
PC(18:0e_20:4)	2.89745	0.00041	***
PC(18:1_18:1)	0.77172	0.03284	*
PC(18:1_18:2)	0.86184	0.15367	
PC(18:1_20:3)	0.69539	0.00239	**
PC(18:1_20:4)	0.81275	0.04738	*
PC(18:1_22:6)	0.62492	0.00751	**
PC(18:1e_16:0)	2.13703	0.00016	***
PC(18:1e_18:1)	3.10757	0.00395	**
PC(18:1e_18:2)	3.57098	0.00001	***
PC(18:1e_20:4)	2.88058	0.00000	***
PC(18:2_18:2)	1.00306	0.98333	
PC(18:2_20:4)	0.78455	0.07165	**
PC(18:2e_18:1)	3.64036	0.00075	***
PC(18:2e_20:4)	4.54856	0.00029	***
PC(20:1_18:2)	1.69164	0.00270	
PC(20:1_20:4)	1.57059	0.00053	***
PC(20:3_20:4)	1.33909	0.02874	*
PC(20:4_20:4)	0.68986	0.01721	*
PC(35:2)	0.46739	0.06243	
PC(35:3)	0.37554	0.02947	*
PE(14:0_18:2)	0.09315	0.00000	***
PE(14:0_20:4)	0.04231	0.00000	***
PE(14:0_22:6)	0.02037	0.00000	***
PE(16:0_16:0)	0.72415	0.03264	*
PE(16:0_16:1)	0.42449	0.00001	***
PE(16:0_18:1)	0.22081	0.00000	***
PE(16:0_18:2)	0.16098	0.00000	***
PE(16:0_20:4)	0.12249	0.00000	***
PE(16:0_20:5)	0.06271	0.00000	***
PE(16:0_22:4)	1.56361	0.00146	**
PE(16:0_22:6)	0.26887	0.00000	***
PE(16:0e_20:4)	0.17636	0.00000	***
PE(16:0e_22:6)	0.71304	0.01618	*
PE(16:1_18:2)	0.20812	0.00000	***
PE(16:1_20:4)	0.17612	0.00000	***
PE(16:1_22:6)	0.17794	0.00000	***
PE(16:1e_18:1)	0.86501	0.53235	
PE(16:1e_18:2)	1.83185	0.02471	*
PE(16:1e_20:4)	0.88033	0.41009	
PE(16:1e_20:5)	0.88251	0.61236	
PE(16:1e_22:4)	1.90470	0.00138	**

PE(16:1e_22:6)	1.53295	0.01848	*
PE(17:0_18:1)	0.34270	0.00000	***
PE(17:0_18:2)	0.35813	0.00000	***
PE(17:0_20:4)	0.20464	0.00000	***
PE(18:0_18:1)	0.69084	0.00661	**
PE(18:0_18:2)	0.47616	0.00001	***
PE(18:0_20:3)	0.89393	0.39125	
PE(18:0_20:4)	0.38910	0.00000	***
PE(18:0_20:5)	0.19217	0.00000	***
PE(18:0_22:4)	2.07647	0.00000	***
PE(18:0_22:5)	2.43056	0.04251	*
PE(18:0_22:6)	1.50518	0.01497	*
PE(18:0e_20:4)	0.30137	0.00000	***
PE(18:1_18:1)	0.34354	0.00000	***
PE(18:1_18:2)	0.30307	0.00000	***
PE(18:1_18:2)	0.25109	0.00000	***
PE(18:1_20:3)	0.43909	0.00000	***
PE(18:1_20:4)	0.23040	0.00000	***
PE(18:1_20:5)	0.28334	0.00000	***
PE(18:1_22:4)	1.39557	0.06954	
PE(18:1_22:5)	0.77740	0.13822	
PE(18:1_22:5)	1.55606	0.25882	
PE(18:1_22:6)	0.50116	0.00003	***
PE(18:1e_18:1)	2.40882	0.02046	*
PE(18:1e_18:2)	3.05423	0.00538	**
PE(18:1e_20:4)	1.43797	0.03699	*
PE(18:1e_20:4)	0.45918	0.00000	***
PE(18:1e_22:6)	1.93828	0.00160	**
PE(18:2_18:2)	0.27308	0.00000	***
PE(18:2_20:4)	0.30978	0.00000	***
PE(18:2_22:6)	0.27284	0.00000	***
PE(18:2e_18:1)	1.41731	0.12596	
PE(18:2e_20:4)	1.65257	0.00398	**
PE(18:2e_22:6)	1.76398	0.00259	**
PE(20:0_20:4)	0.49263	0.00116	**
PE(20:1_18:1)	1.08860	0.55595	
PE(20:1_20:4)	1.22539	0.11534	
PE(20:2_20:4)	0.74571	0.21174	
PE(20:2e_20:4)	2.54535	0.00002	***
PE(20:5_18:2)	0.08491	0.00001	***
PE(32:1e)	2.24717	0.00293	**
PE(32:2e)	3.71435	0.00126	**
PE(34:1e)	0.53801	0.00125	**
PE(38:4e)	1.64379	0.00203	**
PE(38:4e)	2.31058	0.00227	**
PE(38:7e)	1.37767	0.05877	

PE(40:6e)	1.57111	0.00240	**
PE(40:6e)	1.69563	0.00324	**
PE(40:6e)	2.96795	0.00288	**
PE(40:7e)	2.43612	0.00116	**
PE(52:5)	1.10117	0.85792	
PE(54:5)	1.13889	0.80257	
PE(56:5)	1.63020	0.33814	
PG(14:1_14:1)	0.56239	0.20393	
PG(16:0_16:0)	1.02247	0.90816	
PG(16:0_18:1)	0.36795	0.00000	***
PG(16:0_18:2)	0.61453	0.00702	**
PG(16:0_20:2)	0.66603	0.01472	*
PG(16:0_20:4)	0.57530	0.00215	**
PG(16:1_18:1)	2.49311	0.02534	*
PG(16:1_18:2)	1.37678	0.40036	
PG(17:0_17:0)	1.73732	0.01292	*
PG(18:0_18:1)	4.41376	0.01071	*
PG(18:0_18:1)	0.79955	0.15653	
PG(18:0_18:1)	5.96593	0.00676	**
PG(18:0_18:2)	0.70221	0.09562	
PG(18:1_18:1)	4.06408	0.00046	***
PG(18:1_18:2)	1.96075	0.01079	*
PG(18:1_20:2)	3.95860	0.00140	**
PG(18:1_20:4)	1.62495	0.02224	*
PG(18:1_22:5)	1.80892	0.03021	*
PG(18:1_22:6)	0.45989	0.00001	***
PG(18:2_18:2)	1.19016	0.43115	
PG(18:2_20:4)	1.00769	0.96676	
PG(18:2_22:6)	0.33340	0.00000	***
PG(20:3_18:2)	1.04118	0.83835	
PG(20:4_22:6)	0.51781	0.00435	**
PG(22:5_20:4)	1.17270	0.69688	
PG(22:5_22:6)	1.07988	0.88576	
PG(22:6_22:6)	0.19135	0.00000	***
PI(16:0_16:0)	0.46040	0.00038	***
PI(16:0_16:1)	0.94545	0.68710	
PI(16:0_18:1)	0.82060	0.09318	
PI(16:0_18:2)	0.99001	0.93234	
PI(16:0_20:3)	0.70023	0.00610	**
PI(16:0_20:4)	0.60259	0.00001	***
PI(16:0_22:4)	1.66347	0.00201	**
PI(16:0_22:6)	0.42721	0.00000	***
PI(17:0_20:4)	0.74934	0.00426	**
PI(18:0_16:0)	0.24711	0.00000	***
PI(18:0_18:0)	1.37101	0.51366	
PI(18:0_18:1)	0.69727	0.00572	**

PI(18:0_18:2)	1.33893	0.01651	*
PI(18:0_20:1)	0.83736	0.41052	
PI(18:0_20:2)	0.57506	0.00157	**
PI(18:0_20:3)	0.81951	0.09710	
PI(18:0_20:4)	0.98074	0.83271	
PI(18:0_22:4)	2.35725	0.00000	***
PI(18:0_22:5)	2.38754	0.00000	***
PI(18:0_22:5)	1.35526	0.46939	
PI(18:0_22:6)	0.98636	0.94761	
PI(18:1_18:1)	1.45891	0.01352	*
PI(18:1_18:2)	1.07810	0.56923	
PI(18:1_20:3)	1.17858	0.26557	
PI(18:1_20:4)	1.02382	0.83442	
PI(18:1_22:6)	0.46426	0.00009	***
PI(18:1e_20:4)	2.04009	0.00002	***
PI(18:2_18:2)	0.81906	0.32753	
PI(18:2_20:4)	0.80053	0.08078	
PI(36:4e)	0.67013	0.01142	*
PS(16:0_18:1)	0.27255	0.00000	***
PS(16:0_18:2)	0.27293	0.00000	***
PS(16:0_20:4)	0.06317	0.00000	***
PS(18:0_18:1)	0.93712	0.55538	
PS(18:0_18:1)	0.20045	0.00291	**
PS(18:0_18:2)	1.28045	0.04797	*
PS(18:0_20:1)	0.57499	0.14781	
PS(18:0_20:3)	1.23147	0.09539	
PS(18:0_20:4)	0.52585	0.00000	***
PS(18:0_22:4)	0.35418	0.01250	*
PS(18:0_22:5)	2.43698	0.00000	***
PS(18:0_22:6)	1.77235	0.00008	***
PS(18:1_18:1)	1.04603	0.74430	
PS(18:1_20:4)	0.42225	0.00000	***
PS(18:1e_20:4)	0.75373	0.41176	
PS(18:2_18:2)	0.21234	0.00000	***
PS(20:1_20:4)	0.31698	0.00424	**
PS(20:1e_20:4)	1.32608	0.40754	
PS(20:2e_20:4)	1.37711	0.35760	
PS(38:4)	0.11885	0.00227	**
PS(40:6)	0.28064	0.01518	*
PS(42:4)	0.91601	0.78516	
PS(42:7)	0.47405	0.06589	
PS(42:8)	0.61051	0.00008	***
SM(d32:1)	1.25461	0.32859	
SM(d33:1)	1.01608	0.93036	
SM(d34:0)	1.32996	0.19508	
SM(d34:1)	1.10436	0.42296	

SM(d34:1)	0.91975	0.67485	
SM(d34:1)	0.68917	0.62259	
SM(d34:2)	1.25141	0.33480	
SM(d35:1)	1.01224	0.93868	
SM(d35:1)	1.11610	0.51603	
SM(d36:0)	0.65764	0.01110	*
SM(d36:1)	0.66042	0.00342	**
SM(d36:2)	0.64774	0.00716	**
SM(d38:1)	0.58099	0.00121	**
SM(d38:2)	0.61563	0.00206	**
SM(d39:1)	0.72917	0.04517	*
SM(d40:1)	0.70095	0.00826	**
SM(d40:2)	0.73786	0.02538	*
SM(d40:2)	1.25515	0.19692	
SM(d40:3)	1.46361	0.10615	
SM(d41:2)	0.94348	0.68984	
SM(d41:2)	1.16697	0.45037	
SM(d42:1)	1.59545	0.43361	
SM(d42:2)	0.76902	0.09531	
SM(d42:3)	1.17314	0.36377	
SM(d42:4)	1.21851	0.41255	
SM(d43:1)	0.66885	0.01185	*
SM(d44:1)	0.76354	0.05652	
SM(t34:1)	4.48666	0.00016	***
SM(t40:0)	0.09252	0.00000	***

Table S4. Lipid molecules detected by LIMS in healthy and ccRCC samples.

Lipid molecule	ccRCC/healthy (intensity ratio)	T-test	significance
HexCer d28:0	1.52920	0.0001	***
HexCer d29:0	2.91270	0.0003	***
HexCer d30:0	0.89864	0.6302	
HexCer d30:1	1.49482	0.0044	***
HexCer d32:0	0.26802	0.0043	***
HexCer d34:1	1.27622	0.3043	
HexCer d36:1	1.55562	0.0009	***
HexCer d36:2	2.03314	0.0012	***
HexCer d38:2	1.35712	0.1619	
HexCer t33:1	0.97465	0.8490	
HexCer t35:1	3.07106	0.0000	***
HexCer t35:2	3.02447	0.0000	***
HexCer t39:4	5.35089	0.0000	***
Lyso-PI 16:0	1.04577	0.8523	
Lyso-PI 18:0	1.27356	0.0192	*
Lyso-PI 18:1	0.41072	0.0233	*
Lyso-PI 20:4	1.34457	0.2695	
Lyso-PI O-16:2	0.85426	0.5282	
Lyso-PI O-18:2	1.67295	0.0001	***
PC 32:0	1.57919	0.0179	*
PC 32:2	0.34534	0.0002	***
PC 33:2	0.89077	0.5192	
PC 34:1	0.80361	0.0056	**
PC 34:4	0.15894	0.0000	***
PC 35:1	0.60786	0.0306	*
PC 35:2	1.60156	0.0144	*
PC 36:1	0.76780	0.0929	
PC 36:2	1.71238	0.0000	***
PC 36:5	0.00000	0.0000	
PC 36:6	0.30063	0.0007	***
PC 38:4	1.32067	0.0908	
PC 38:5	1.45823	0.0008	***
PC 38:6	1.63214	0.0046	***
PC 38:7	7.30906	0.0223	*
PC O-32:2	0.97465	0.8490	
PC O-34:2	3.07106	0.0000	***
PC O-34:3	3.02447	0.0000	***
PC O-34:5	1.14381	0.0737	
PC O-36:5	2.12307	0.0000	***
PC O-36:6	2.78241	0.0000	
PC O-36:7	2.37120	0.0000	***
PC O-38:5	5.35089	0.0000	***
PC O-38:6	8.21934	0.0000	***
PC O-38:7	0.00000	0.0000	***

PE 34:0	1.57919	0.0179	*
PE 34:1	0.67739	0.0002	***
PE 34:2	0.34534	0.0002	***
PE 35:2	0.89077	0.5192	
PE 36:1	0.80361	0.0056	**
PE 36:2	0.96205	0.6843	
PE 36:3	0.65540	0.0008	***
PE 36:4	0.15894	0.0000	***
PE 37:1	0.60786	0.0306	*
PE 37:2	1.60156	0.0144	*
PE 38:1	0.76780	0.0929	
PE 38:2	1.71238	0.0000	***
PE 38:4	0.67230	0.0001	***
PE 38:5	0.00000	0.0000	
PE 38:6	0.30063	0.0007	***
PE 40:4	1.32067	0.0908	
PE 40:5	1.45823	0.0008	***
PE 40:6	1.63214	0.0046	***
PE 40:7	7.30906	0.0223	*
PE O-34:2	0.97465	0.8490	
PE O-34:3	3.09989	0.0002	***
PE O-36:2	3.07106	0.0000	***
PE O-36:3	3.02447	0.0000	***
PE O-36:5	1.14381	0.0737	
PE O-38:5	2.12307	0.0000	***
PE O-38:6	2.78241	0.0000	
PE O-38:7	2.37120	0.0000	***
PE O-40:5	5.35089	0.0000	
PE O-40:6	8.21934	0.0000	
PE O-40:7	0.00016	0.0000	***
PG 40:7	1.21336	0.6545	
PG 24:5	0.00000	0.0000	***
PG 34:1	0.41741	0.0000	***
PG 36:1	0.14653	0.0000	***
PG 36:2	0.20315	0.0000	
PG 38:2	0.00037	0.0000	***
PG 39:5	0.10701	0.0009	***
PG 41:5	0.01286	0.0008	***
PG 41:6	2.88677	0.0000	***
PG 43:6	7.12347	0.0000	***
PG 44:5	0.09319	0.0010	***
PG 44:9	0.00000	0.0003	***
PG 45:5	0.00641	0.0000	***
PG 46:5	0.00000	0.0001	***
PG 46:6	0.00000	0.0004	***
PG O-35:4	0.00000	0.0000	***

PG O-38:6	0.35210	0.0103	*
PI 32:0	0.22002	0.0000	***
PI 34:1	1.00725	0.9440	
PI 34:2	0.00000	0.0000	
PI 36:1	0.86510	0.2732	
PI 36:2	1.62925	0.0000	***
PI 36:3	0.00000	0.0000	***
PI 36:4	0.00000	0.0000	
PI 38:2	0.54884	0.0506	
PI 38:4	1.28706	0.0007	***
PI 38:5	1.12685	0.3190	
PI 38:6	0.50897	0.0112	*
PI 40:4	3.66103	0.0000	***
PI 40:5	2.11785	0.0188	*
PI 40:6	1.47685	0.1616	
PS 34:1	0.06890	0.0000	***
PS 36:1	0.86615	0.3962	
PS 36:2	0.00000	0.0000	***
PS 36:4	0.03497	0.0000	***
PS 38:3	1.41606	0.2575	
PS 38:4	0.99947	0.9958	
PS 38:5	1.78468	0.0278	*
PS 40:7	0.76663	0.3149	
PS O-38:2	0.92263	0.6159	
SM d32:1	1.15801	0.4481	
SM d33:1	0.83268	0.4689	
SM d34:1	0.88586	0.3854	
SM d34:2	1.13148	0.5095	
SM d35:1	1.01739	0.9384	
SM d36:1	0.52684	0.0001	***
SM d36:2	0.53887	0.0346	*
SM d38:1	0.47068	0.0039	***
SM d38:2	0.58891	0.0984	
SM d40:1	0.64112	0.0007	***
SM d40:2	0.00000	0.0000	***
SM d42:1	0.04595	0.0000	***
SM d42:2	1.31930	0.0119	*
SM d42:3	0.96671	0.8583	
SM t32:2	1.05001	0.6491	
Sulfatide d34:1	2.21782	0.1540	
Sulfatide d34:2	0.00000	0.0018	***
Sulfatide d34:3	0.00000	0.0001	***
Sulfatide d36:3	0.85104	0.5770	
Sulfatide d36:4	5.13728	0.0109	*
Sulfatide d38:1	0.68216	0.4246	
Sulfatide d40:1	0.09892	0.0002	***

Sulfatide d40:2	1.82250	0.0856	
Sulfatide d41:1	0.07915	0.0006	***
Sulfatide d41:2	0.00000	0.0004	***
Sulfatide d42:1	0.52578	0.0622	
Sulfatide d42:2	1.96476	0.0027	***
Sulfatide d42:3	8.93577	0.0015	***
Sulfatide d43:3	0.77551	0.3491	
Sulfatide t35:0	0.99947	0.9958	
Sulfatide t41:0	0.00000	0.0192	*
Sulfatide t41:1	0.04156	0.0012	***
Sulfatide t42:0	0.11898	0.1504	
Sulfatide t42:1	0.14613	0.0134	*
